# Identification of *MYCN* non-amplified neuroblastoma subgroups points towards molecular signatures for precision prognosis and therapy stratification

**DOI:** 10.1038/s41416-024-02666-y

**Published:** 2024-03-29

**Authors:** Xiaoxiao Hu, Yilu Zhou, Charlotte Hill, Kai Chen, Cheng Cheng, Xiaowei Liu, Peiwen Duan, Yaoyao Gu, Yeming Wu, Rob M Ewing, Zhongrong Li, Zhixiang Wu, Yihua Wang

**Affiliations:** 1 Department of Paediatric Surgery, Xinhua Hospital, School of Medicine, Shanghai Jiaotong University, Shanghai 200092, China; 2 Department of Paediatric Surgery, The Second Affiliated Hospital and Yuying Children's Hospital of Wenzhou Medical University, Wenzhou 325000, China; 3 Division of Paediatric Oncology, Shanghai Institute of Paediatric Research, Shanghai 200092, China; 4 Biological Sciences, Faculty of Environmental and Life Sciences, University of Southampton, Southampton SO17 1BJ, UK; 5 Institute for Life Sciences, University of Southampton, Southampton SO17 1BJ, UK; 6 Department of Paediatric Surgery, Children's Hospital of Soochow University, Suzhou 215003, China

## Abstract

**Background:**

Despite the extensive study of *MYCN*-amplified neuroblastomas, there is a significant unmet clinical need in *MYCN* non-amplified cases. In particular, the extent of heterogeneity within the *MYCN* non-amplified population is unknown.

**Methods:**

A total of 1,566 samples from 16 datasets were identified in Gene Expression Omnibus (GEO) and ArrayExpress. Characterisation of the subtypes was analysed by ConsensusClusterPlus. Independent predictors for subgrouping were constructed from the single sample predictor based on the multiclassPairs package. Findings were verified using immunohistochemistry and CIBERSORTx analysis.

**Results:**

We demonstrate that *MYCN* non-amplified neuroblastomas are heterogeneous and can be classified into 3 subgroups based on their transcriptional signatures. Within these groups, subgroup_2 has the worst prognosis and this group shows a "*MYCN*" signature that is potentially induced by the overexpression of Aurora Kinase A (AURKA); whilst subgroup_3 is characterised by an "inflamed" gene signature. The clinical implications of this subtype classification are significant, as each subtype demonstrates a unique prognosis and vulnerability to investigational therapies. A total of 420 genes were identified as independent subgroup predictors with average balanced accuracy of 0.93 and 0.84 for train and test datasets, respectively.

**Conclusion:**

We propose that transcriptional subtyping may enhance precision prognosis and therapy stratification for patients with *MYCN* non-amplified neuroblastomas.

## Introduction

Neuroblastoma is the most common extra-cranial solid tumour in children, representing 6-10% of all childhood cancers^1^. It is an embryonic tumour arising from precursor cells in the sympathetic nervous system and adrenal medulla^2^, with a median age of diagnosis of 18 months^3^. It can also be present in the neck, chest, abdomen, or pelvis^4^. Neuroblastoma is a highly heterogeneous disease, with clinical behaviour ranging from spontaneous regression to drug resistance and metastasis ultimately resulting in death^5^. The prognosis of the disease is poor with a 5-year overall survival of approximately 20%, despite more aggressive therapies^6^. As a result, risk stratification and personalised treatment approaches in neuroblastomas are urgently needed.

The International Neuroblastoma Risk Group Staging System (INRGSS) defines the high-risk group to include patients with *MYCN*-amplified tumours and patients > 18 months old with metastatic tumours^7^. N-MYC is a key regulator of transcription, which activates genes that affect cancer development. It is widely involved in various pathological processes of neuroblastoma including cell growth^8^, apoptosis^9^, differentiation^10^, angiogenesis^11^, tumour invasion, and metastasis^12^.

*MYCN* amplification was identified as the first independent prognostic factor indicating adverse clinical outcomes in neuroblastomas^13,14^, which is observed in approximately 20% of cases^15^ and accounts for about 40% of high-risk neuroblastomas^16^. Despite the extensive study of *MYCN*-amplified neuroblastomas, there is a significant unmet clinical need in *MYCN* non-amplified cases. In particular, the extent of heterogeneity within the *MYCN* non-amplified population is unknown.

Here, we investigated whether transcriptional subtyping of *MYCN* non-amplified neuroblastomas can identify molecular subtypes with discrete prognosis and therapeutic vulnerabilities. Our analysis suggested that *MYCN* non-amplified neuroblastomas were heterogeneous and could be classified into 3 subgroups based on their transcriptional profiling. Within them, subgroup 2 had the worst prognosis and this group had a "*MYCN*" signature that was potentially induced by the overexpression of Aurora Kinase A (AURKA); whilst subgroup 3 was accompanied by an "inflamed" gene signature. We propose that transcriptional subtyping may enhance precision prognosis and therapy stratification for patients with *MYCN* non-amplified neuroblastomas.

## Results

### Characterisation of molecular subtypes in *MYCN* non-amplified neuroblastomas

**Figure 1:**
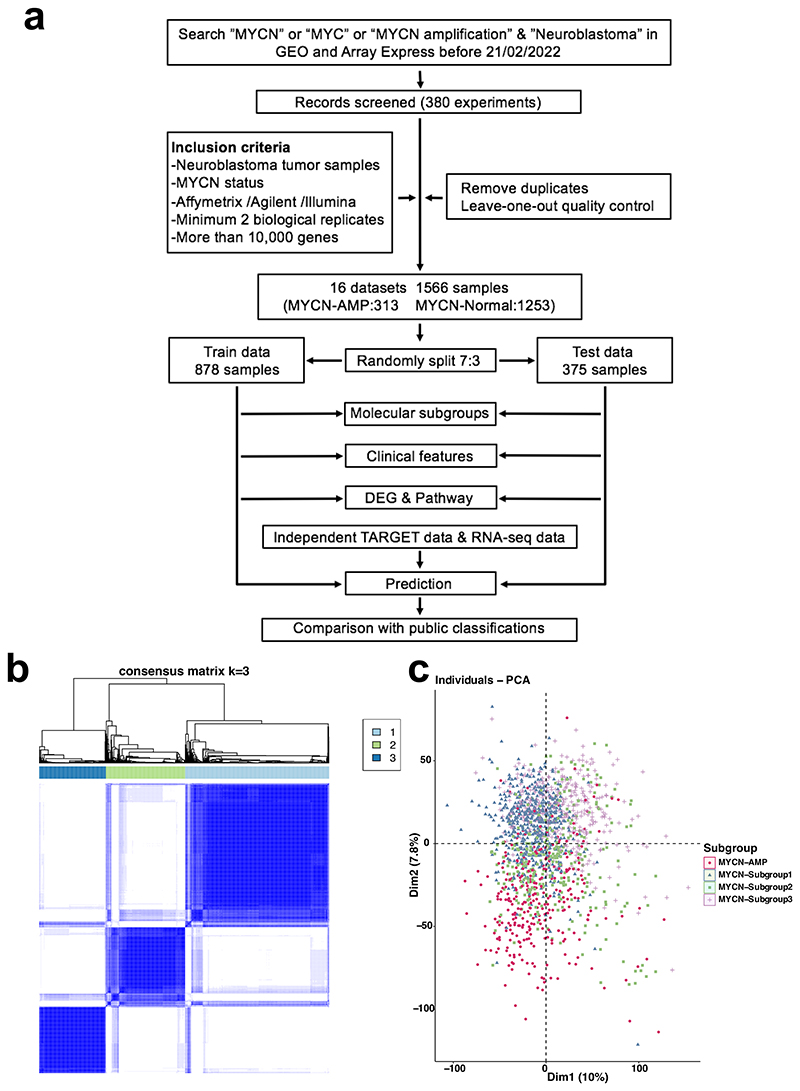
Characterisation of molecular subtypes in the *MYCN* non-amplified neuroblastomas. (**a**) Workflow showing the study design (details provided in the Supplementary Methods). (**b**) Consensus clustering of top 50% variable genes of train cohort. (**c**) Principal component analysis (PCA) showing neuroblastoma patients with subgroup annotations.

Following quality control and eliminating duplicates (Supplementary Figs. 1 and 2; details provided in the Supplementary Methods), a total of 1,566 samples from 16 datasets were identified in GEO (Gene Expression Omnibus) and ArrayExpress, in which 313 cases are with *MYCN* gene amplification (*MYCN*-AMP) and 1,253 cases *MYCN* non-amplified (*MYCN*-normal) (Fig. 1a; Supplementary Table 1). Following the removal of batch effects (Supplementary Fig. 3a), 2 clear clusters corresponding to *MYCN*-AMP and *MYCN*-normal neuroblastomas, respectively, were visualised using principal component analysis (PCA) (Supplementary Fig. 3b). Samples in the *MYCN*-normal group (n = 1,253) were further randomly divided into a train and a test group with a 7:3 ratio, containing 878 and 375 cases, respectively (Fig. 1a).

In an unbiased attempt to identify subtypes within *MYCN* non-amplified neuroblastomas, we applied consensus clustering to both train and test groups based on 5,792 variable genes (top 50% median absolute deviation; Supplementary Table 2). As determined by the relative area under the cumulative distribution function and cluster-consensus scores, the optimal number of distinct clusters was 3 (Fig. 1b; Supplementary Fig. 3c). In total, within the *MYCN* non-amplified group, subgroup 1 (blue), 2 (green) and 3 (purple) accounts for 46%, 30%, and 24%, respectively (Fig. 1c). Cross-cohort analysis using an unsupervised method SubMap^17^ (https://www.genepattern.org/modules) confirmed the robustness of this classification (Supplementary Fig. 4a; false discovery rate, FDR < 0.05).

**Figure 2:**
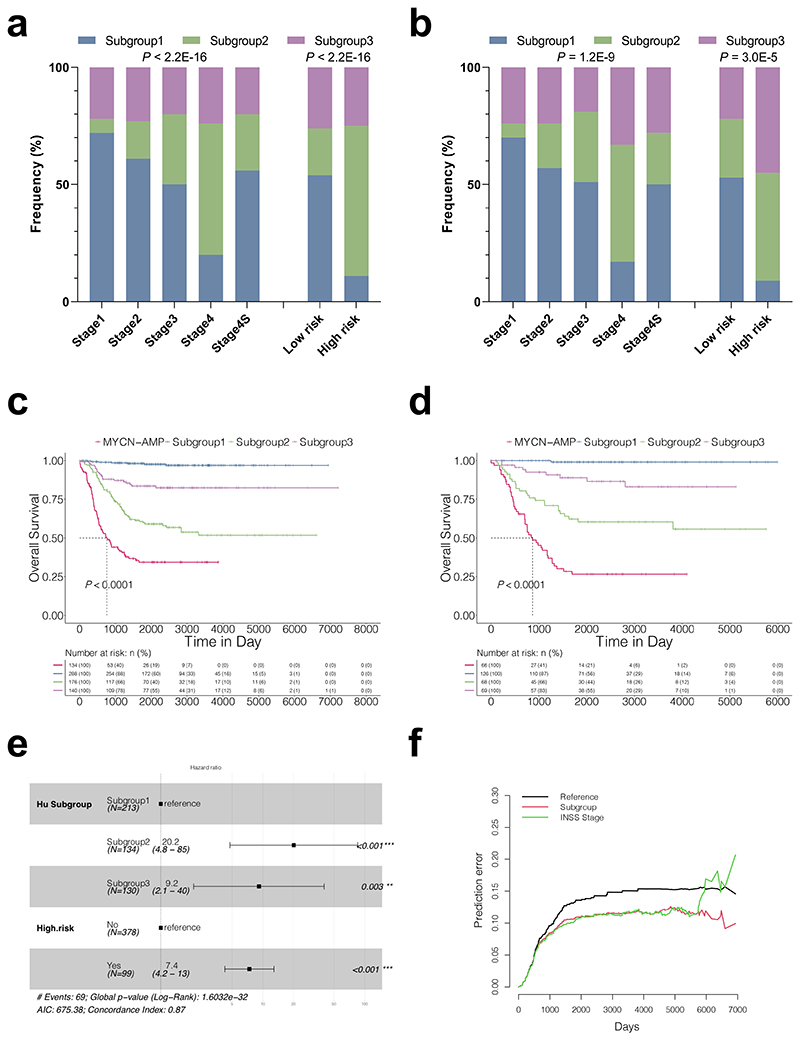
Clinical characterisation of subtypes within *MYCN* non-amplified neuroblastomas identifies key distinguishing features. Graphs showing the frequency (%) of each molecular subtype in different International Neuroblastoma Staging System (INSS) stages or risk status in either train (**a**) or test (**b**) cohort. *P* values are indicated. Kaplan-Meier plots showing the overall survival in each molecular subtype or *MYCN*-amplification (*MYCN*-AMP) in either train (**c**) or test (**d**) cohort. The numbers below are n (%). P values are indicated. (**e**) Multivariate analysis of subgroup classification with risk status in *MYCN* non-amplified neuroblastomas. HR (hazard ratio), 95% CI (confidence interval), patient number (n), and *P* values are shown. (**f**) Prediction error curves (indicating a mean squared error in predicting survival status) are calculated for the subgroup (red) and INSS stage (green).

Further clinical characterisation of these subtypes identified key distinguishing features. Patients within subgroup 2 were frequently observed in the advanced neuroblastomas according to the International Neuroblastoma Staging System (INSS) and in those defined as "high risk"^7^ (Fig. 2a and b; Supplementary Fig. 4b). We then analysed their overall survival together with *MYCN*-AMP cases. Patients with *MYCN* amplification had the worst prognosis (Fig. 2c and d; Supplementary Fig. 4c). Importantly, there was a high degree of variability for overall survival among *MYCN* non-amplified cases, in which subgroup 2 was associated with a poor prognosis, followed by subgroup 3; while patients within subgroup 1 had the most favourable outcomes. These observations were consistent in both train and test cohorts. In addition, the molecular subtype classification was a strong independent predictor of mortality including in multivariate analysis with the risk classification that uses commonly measured clinical variables to predict mortality in neuroblastomas^7^. Using subgroup 1 as a reference, the hazard ratio (HR) and 95% confidence interval (CI) for subgroups 2 and 3 were 20.2 (4.8 ∼ 85) and 9.2 (2.1 ∼ 40), respectively (Fig. 2e). Similar results were obtained using univariate or multivariate cox regression analysis with age and INSS stages in *MYCN* non-amplified neuroblastomas (Supplementary Table 3). A comprehensive multivariate analysis also revealed our subgroups to be independent of genomic features such as 1p, 11q, and 17q (Supplementary Fig. 4d-f)). Impressively, the molecular subtype classification alone outperformed INSS stages (Fig. 2f) and shows a comparable prediction accuracy as the risk classification (Supplementary Fig. 4g).

Overall, subgroup 2 and subgroup 3 (to a lesser extent) were associated with poor survival in *MYCN* non-amplified neuroblastomas, suggesting fundamentally different mechanisms leading to an advanced disease.

### Defining molecular features of the 3 subtypes in *MYCN* non-amplified neuroblastomas

**Figure 3:**
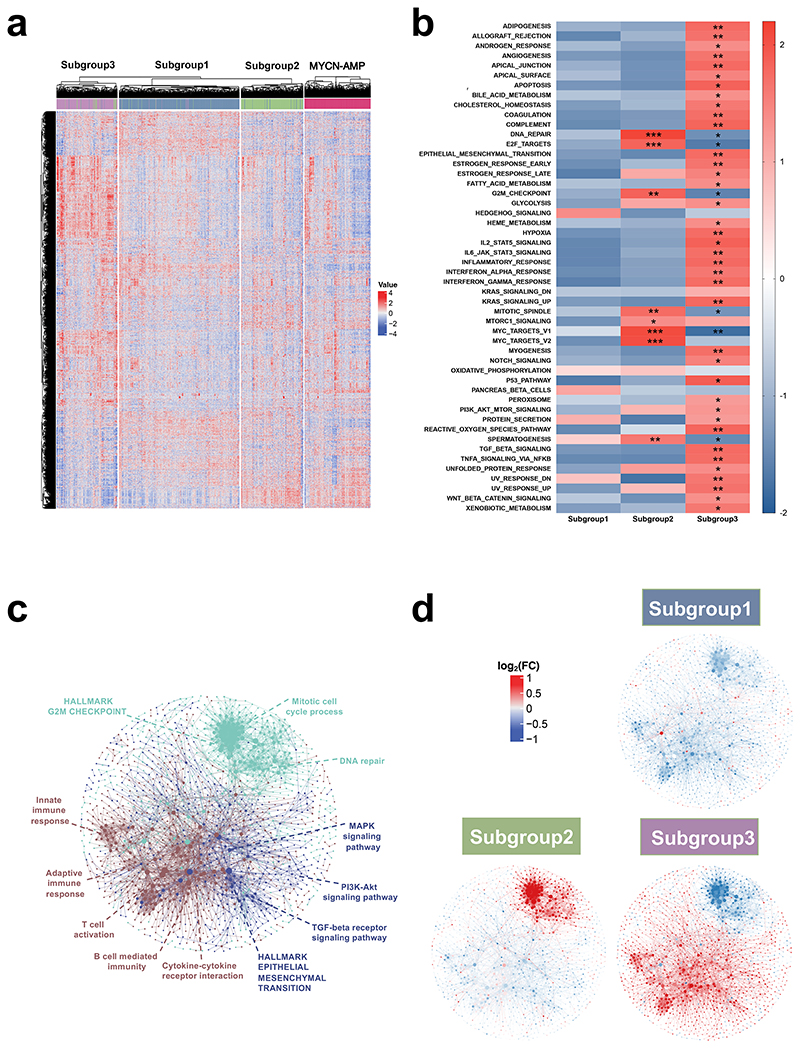
Defining molecular features of 3 subtypes in *MYCN* non-amplified neuroblastomas. (**a**) Heatmap showing differential expression of selected genes. Red indicates up-regulation and blue for down-regulation. Colour bars show subgroup information. (**b**) Gene set enrichment analysis (GSEA) in 3 subtypes. *FDR (false discovery rate) < 0.25; **FDR < 0.05; ***FDR < 0.01. (**c**) Weighted gene co-expression network analysis (WGCNA) showing 3 molecular modules. Nodes are colour-coded according to the WGCNA modules. Representative enriched pathway terms are indicated. (**d**) Overlay of the median-cantered log_2_ fold change values per subgroup on the network.

Using the same 5,792 variable genes described above (Supplementary Table 2), we observed clear distinctions among these 3 subtypes in *MYCN* non-amplified neuroblastomas (Fig. 3a; Supplementary Table 4). Intriguingly, subgroup 2 showed a similar signature to *MYCN*-AMP cases (Fig. 3a). This was consistent with the Gene Set Enrichment Analysis (GSEA), showing HALLMARK_MYC_TARGETS_V1 and V2 significantly enriched in subgroup 2 (Fig. 3b; Supplementary Table 5; FDR = 0.0021 and 0.0017, respectively). In contrast, subgroup 3 exhibited an "inflamed" phenotype, with high expression of genes related to IL6_JAK_STAT3_SIGNALING, INFLAMMATORY_RESPONSE, INTERFERON_ALPHA_RESPONSE and INTERFERON _GAMMA_RESPONSE (Fig. 3b; Supplementary Table 5; all FDR values less than 0.05). None of these pathways were enriched in subgroup 1 (Fig. 3b).

The above analysis was extended using weighted gene co-expression network analysis (WGCNA)^18^. Three molecular modules were identified (Supplementary Fig. 5; Supplementary Table 6) and were further used to construct a protein-protein network consisting of 1,393 genes and 4,490 edges (Fig. 3c; confidence score > 0.9). Molecular module MEturquoise, which was significantly correlated with subgroup 2 (Fig. 3d), was enriched for "Mitotic cell cycle process", "HALLMARK G2M CHECKPOINT", and " DNA repair". In subgroup 3, there were 2 molecular modules, MEblue and MEbrown highly involved (Fig. 3d; Supplementary Table 6). Molecular module MEblue was enriched for pathways, including "HALLMARK EPITHELIAL MESENCHYMAL TRANSITION", "TGF-beta receptor signaling pathway", "PI3K-Akt signaling pathway" and " MAPK signaling pathway" whereas " Cytokine-cytokine receptor interaction", "T cell activation", "B cell-mediated immunity", "Adaptive immune response" and "Innate immune response" were significantly enriched in molecular module MEbrown.

### Subgroup 2 shows a "*MYCN*" signature, potentially induced by Aurora Kinase A (AURKA) overexpression

**Figure 4:**
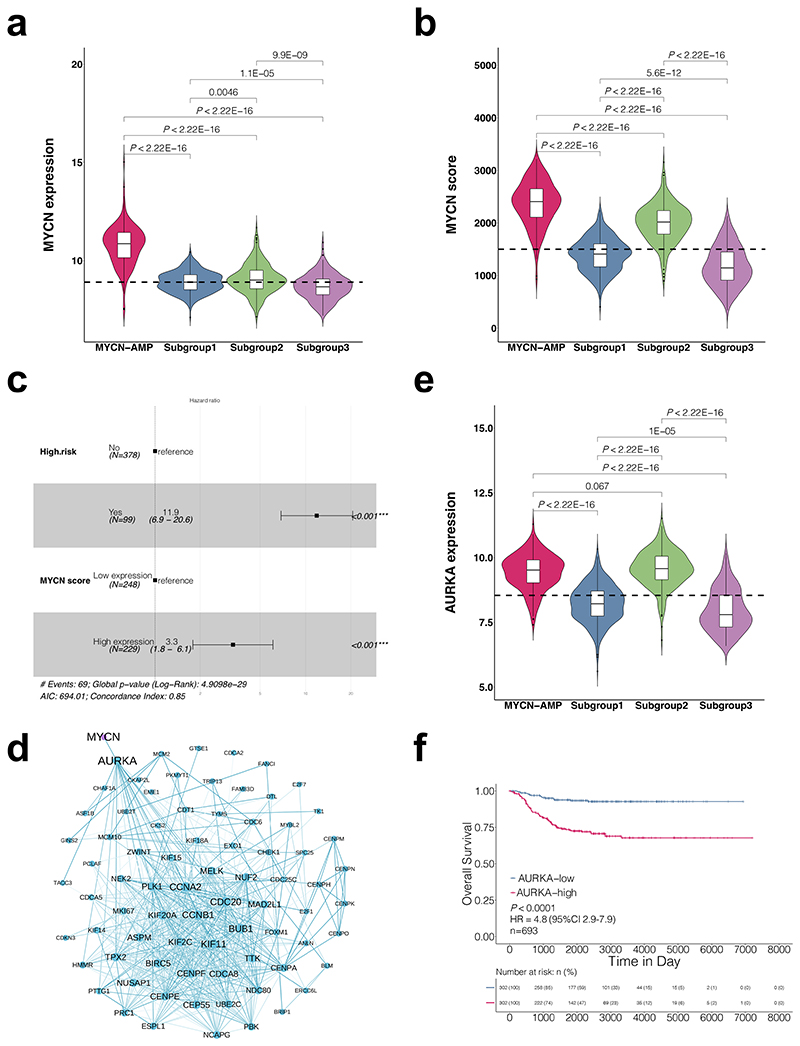
Subgroup 2 shows a "*MYCN*" signature, potentially induced by Aurora Kinase A overexpression. Violin plots showing *MYCN* mRNA levels (**a**) or *MYCN* scores (**b**) in neuroblastomas. *P* values are indicated. (**c**) Multivariate analysis of *MYCN* score and risk status in *MYCN* non-amplified neuroblastomas. HR (hazard ratio), 95% CI (confidence interval), patient number (n), and *P* values are shown. (**d**) Protein-protein interaction (PPI) network showing an interaction between AURKA and MYCN. (**e**) Violin plot showing *AURKA* mRNA levels in neuroblastomas. *P* values are indicated. (**f**) Kaplan-Meier plot showing the overall survival in samples with low *vs*. high *AURKA* expression. The numbers below are n (%). HR (hazard ratio), 95% CI (confidence interval), patient number (n), and *P* values are shown.

Our above analysis suggested that mechanisms other than gene amplification induce N-MYC activity in subgroup 2. Indeed, the mRNA level of *MYCN* in subgroup 2 was significantly lower than cases within the *MYCN*-AMP group (Fig. 4a; Supplementary Table 4; *P* < 0.0001). To evaluate N-MYC activity in neuroblastoma samples, a total of 87 genes upregulated by N-MYC were selected to classify its activity^19^. The *MYCN* score for each sample was calculated using single-sample gene set enrichment analysis (ssGSEA) based on this 87-gene expression signature. *MYCN* scores in subgroup 2 were significantly higher than those in subgroups 1 and 3, and were comparable to those in the *MYCN*-AMP group, although slightly lower (Fig. 4b). Moreover, the *MYCN* score was an independent predictor of mortality including in multivariate analysis with the risk classification (Fig. 4c; HR: 3.3; *P* < 0.001).

To investigate the potential mechanism that leads to higher *MYCN* scores in subgroup 2, correlation analysis coupled with protein-protein interactions (PPI) network construction was performed (Fig. 4d; Supplementary Table 7). Among the candidate genes, *AURKA* (Aurora kinase A) was identified to interact with *MYCN*. AURKA, a serine/threonine kinase regulating the process of mitosis^20^, was previously demonstrated to regulate N-MYC protein stability^21^. *AURKA* was expressed at significantly higher levels in subgroup 2 when compared to the other 2 subgroups and its levels were even slightly higher than those in the *MYCN*-AMP group (Fig. 4e). Classifying *MYCN* non-amplified neuroblastomas into high and low groups, we demonstrated that the *AURKA* mRNA levels alone could predict the overall survival (Fig. 4f; HR 4.8; *P* < 0.0001). In addition, the high level of *AURKA* was an independent predictor (HR 3, *P* < 0.001) of mortality including in multivariate analysis with the risk classification (Supplementary Fig. 6).

**Figure 5:**
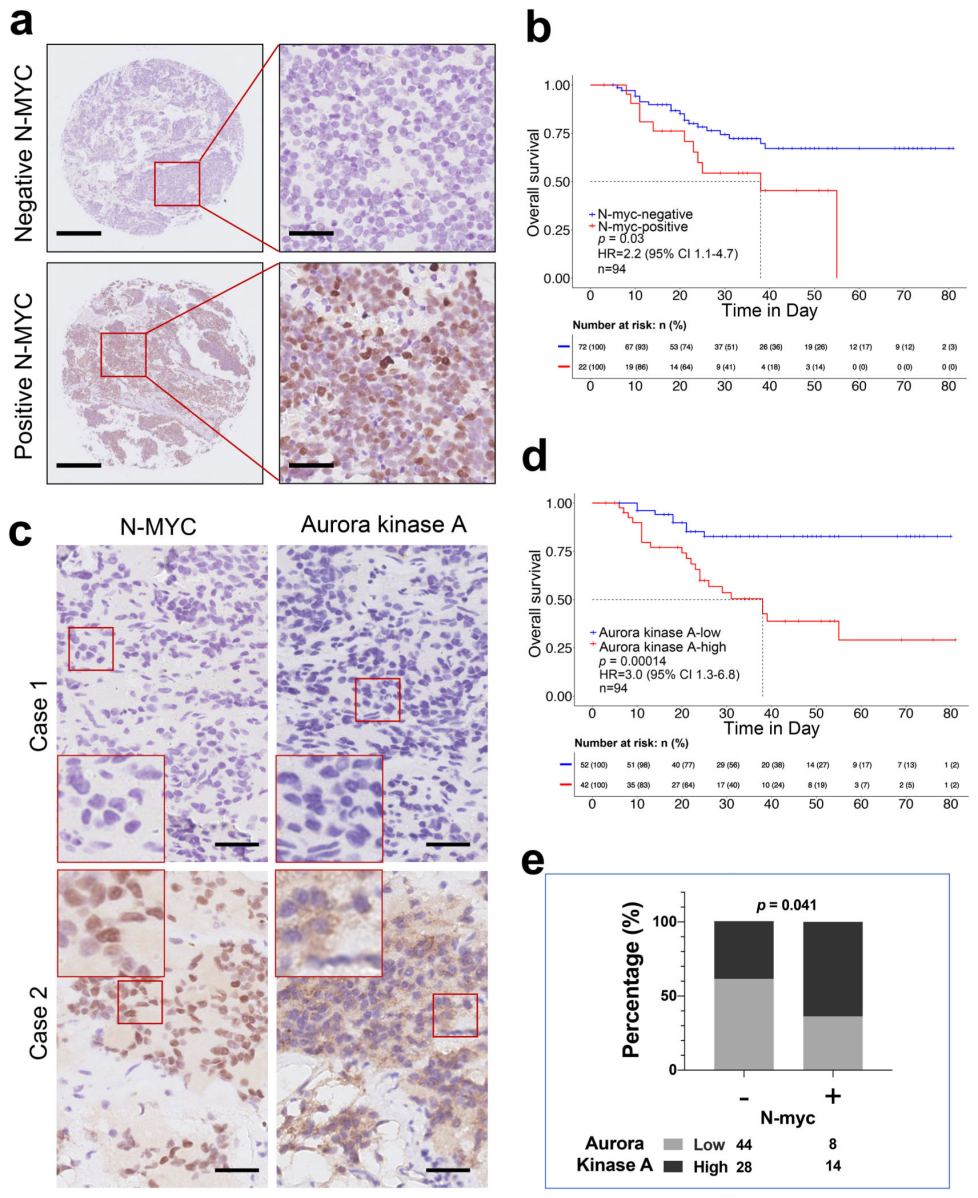
N-MYC expression correlates with Aurora kinase A status in *MYCN* non-amplified neuroblastomas and is indicative of patient survival. (**a**) Representative N-MYC staining pattern (negative or positive N-MYC) in *MYCN* non-amplified neuroblastoma tissue microarray cores. Scale bar: 1mm (the left column) and 50μm (the right column). (**b**) Kaplan-Meier plot showing the overall survival in samples with negative *vs*. positive N-MYC expression. The numbers below are n (%). HR (hazard ratio), 95% CI (confidence interval), patient number (n), and *P* values are shown. (**c**) Adjacent tumour sections from representative cases showing N-MYC and Aurora Kinase A expression in *MYCN* non-amplified neuroblastoma. Scale bars: 50μm. (**d**) Kaplan-Meier plot showing the overall survival in samples with low *vs*. high Aurora kinase A expression. The numbers below are n (%). HR (hazard ratio), 95% CI (confidence interval), patient number (n), and *P* values are shown. (**e**) Graph showing percentage (%) and numbers of samples with low or high Aurora kinase A in the negative or positive N-MYC group. *P* = 0.041.

These findings were further investigated by immunohistochemistry (IHC) staining of N-MYC or AURKA in a custom neuroblastoma tissue microarray, which contains 94 *MYCN* non-amplified neuroblastomas. Within them, 22 samples were positive for N-MYC (Fig. 5a), and they had worse survival compared to those with N-MYC negative staining (n = 72) (Fig. 5b; *P* = 0.03; Supplementary Table 8). In parallel, patients with higher levels of AURKA had unfavourable survival outcomes (Fig. 5c and d; *P* = 0.00014). Moreover, a higher percentage of patients with high AURKA staining was observed in the N-MYC-positive group compared to the N-MYC-negative group (Fig. 5e; 64% *vs*. 39%; *P* = 0.041).

Taken together, these results suggested that a "*MYCN*" signature in subgroup 2 is potentially induced by AURKA overexpression in *MYCN* non-amplified neuroblastomas.

### Subgroup 3 is accompanied by an "inflamed" gene signature

**Figure 6:**
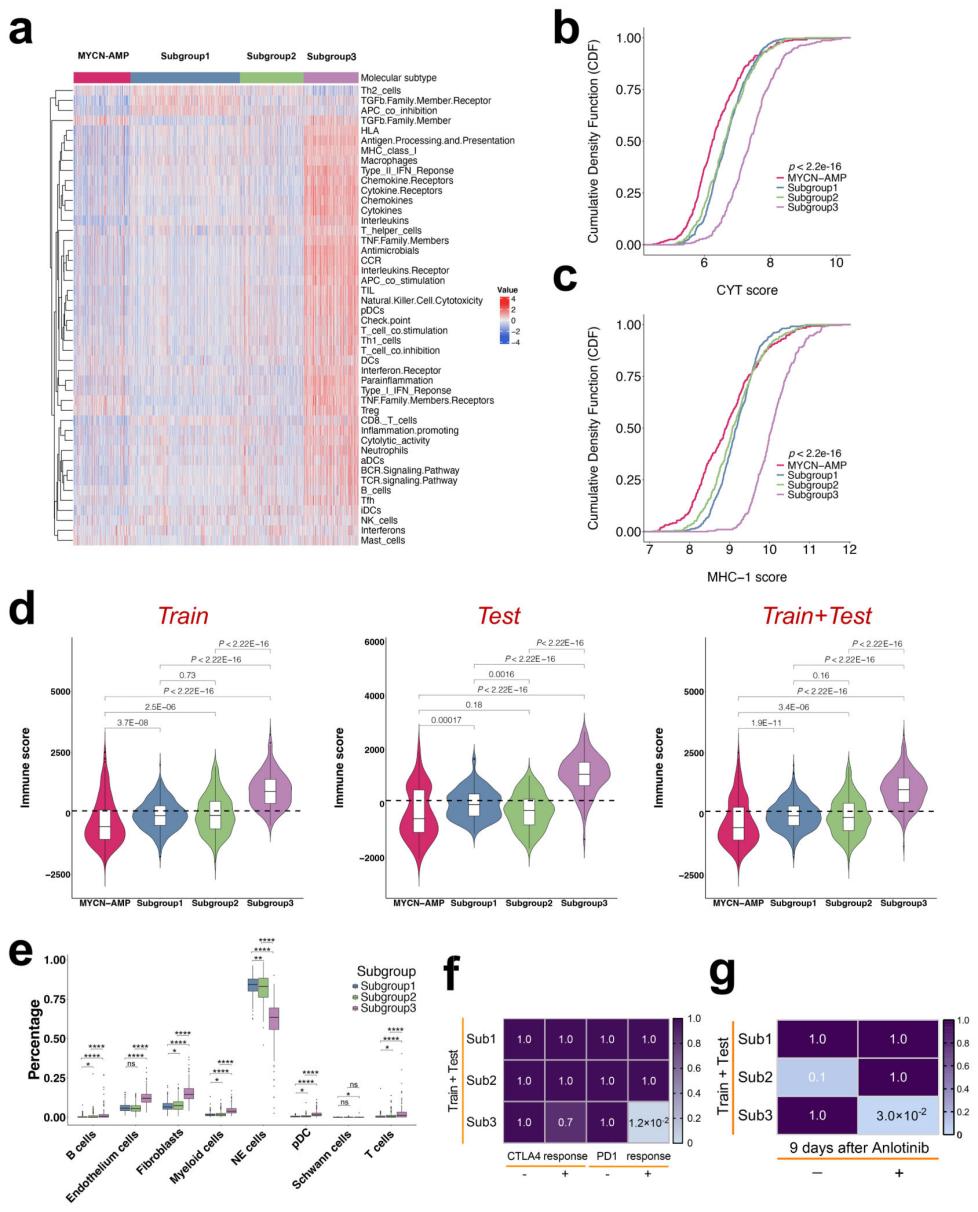
Subgroup 3 is accompanied by an "inflamed" gene signature. (**a**) Heatmap showing neuroblastoma-associated immune pathways and immune cell signatures in subgroups and *MYCN*-AMP. Graphs showing the cumulative distribution of CYT (**b**) or MHC-1 (**c**) scores in different subgroups and *MYCN*-AMP. (**d**) Violin plots showing immune scores in different subgroups and *MYCN*-AMP in train, test, or train plus test cohort. (**e**) Graph showing cell compositions of each subgroup using CIBERSORTx analysis. (**f**) Graph showing differential putative immunotherapeutic response in different subgroups. Bonferroni adjusted *P* values indicated. (**g**) Subclass association (SA) matrix for the comparison between different subgroups and vehicle/anlotinib treated mouse. Bonferroni adjusted *P* values indicated.

Considering immune-related pathways were enriched in subgroup 3, the activity of immune cells and pathways were further systematically explored. ssGSEA was performed to calculate enrichment scores of 46 immune gene sets summarised from two previous studies^22,23^, and subgroup 3 showed significantly higher activity of immune cells and pathways compared to the other 2 subtypes as well as *MYCN*-AMP group (Fig. 6a; Supplementary Table 9). Consistently, cytolytic activity (CYT) or MHC-1 (major histocompatibility complex-1) scores were highest in subgroup 3 (Fig. 6b and c). This was also true when using the ESTIMATE algorithm to evaluate the immune scores, stromal scores, and tumour purity scores in neuroblastomas^24^, showing the highest immune and stromal scores, and lowest tumour purity scores in subgroup 3 (Fig. 6d; Supplementary Fig. 7a and b).

For a comprehensive assessment of immune cell infiltration, we used CIBERSORTx deconvolution^25^ to quantify various immune populations based on a single cell RNA sequencing (scRNA-seq) dataset in *MYCN* non-amplified neuroblastoma^26^ (Supplementary Fig. 7c). While similar immune cell types were present in each subtype, the absolute number of several immune cell populations were markedly increased in subgroup 3, including B cells, myeloid cells, T cells and pDC (plasmacytoid dendritic cells) (Fig. 6e). Finally, to investigate whether subgroup 3 would benefit more from immunotherapy than the other subgroups, we compared the expression matrix of 3 subgroups with published melanoma datasets including response information after treating with immunotherapies^27,28^. The SubMap analysis highlighted that patients within subgroup 3 are predicted to respond to anti-PD1 immunotherapy (Fig. 6f; Supplementary Fig. 7d). In addition, Su *et al*. observed that anlotinib treatment in neuroblastoma mice reprogrammed the immunosuppressive tumour microenvironment (TME) into an immune-stimulatory TME, leading to an extension in the duration of vascular normalization, and dynamic changes in the expression levels of PD-1 and PD-L1. In addition, it is noteworthy that the combination of anlotinib with PD-1 checkpoint inhibitors counteracted the immune suppression induced by PD-L1 upregulation after monotherapy, ultimately inducing the regression of neuroblastoma^29^. Therefore, we reanalysed the RNA-seq data of neuroblastoma syngeneic mouse models treated with vehicle/anlotinib for 9 days. Then, we compared the molecular features of each condition to our subgroups. Interestingly, SubMap analysis revealed that subgroup 3 exhibited a significant similarity in expression profile to mouse models after anlotinib treatment (Fig. 6g; *P* = 0.032).

Taken together, these results demonstrated that subgroup 3 is accompanied by an "inflamed" gene signature, and is more likely to benefit from anti-PD1 therapies.

### Identification of independent predictors to subgroup patients within *MYCN* non-amplified neuroblastomas

To identify independent predictors for subgrouping, we applied a multi-cohort analysis pipeline via multiclassPairs^30^ (see Supplementary Methods). In total, a random forest model, trained using 928 rules derived from a set of 432 genes (Supplementary Table 10) displayed the ability to predict different subgroups accurately in both training and test sets with an F1 score > 0.74 (Supplementary Table 11). The prediction model and example files can be downloaded from https://zenodo.org/records/10258748.

**Figure 7:**
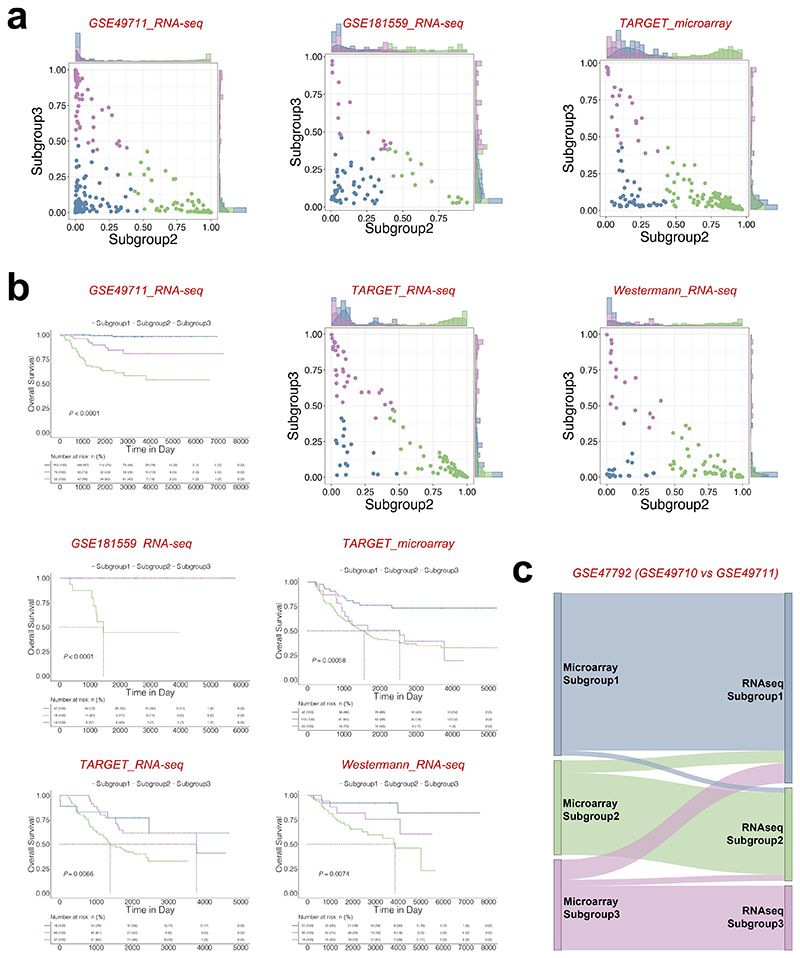
Identification and evaluation of independent predictors to subgroup patients within *MYCN* non-amplified neuroblastomas. (**a**) Predicted probability of each subgroup in 5 different cohorts. Each dot in the scatter plot corresponds to a sample (x-axis: predicted probability of subgroup 2, y-axis: predicted probability of subgroup 3). The histogram plot above the scatter plot displayed the distribution of subgroup 2 probabilities while the plot to the right of the scatter plot displayed the distribution of subgroup 3 probabilities. (**b**) Kaplan-Meier plots showing the overall survival in predicted molecular subtype in 5 different cohorts. The numbers below are n (%). *P* values are indicated. (**c**) Prediction differences in the superseries GSE47792 using data from either RNA-seq (GSE49711) or microarray (GSE49710).

Furthermore, the random forest model successfully stratified patients with *MYCN* non-amplified neuroblastoma into distinct subgroups 1, 2, and 3 with significant differences in survival across five independent validation sets (GSE49711^31^, TARGET Microarray^32^, TARGET RNA-seq, Westermann ALK cohort^33^ and Stefan Hüttelmaier cohort^34^ respectively) (Fig. 7a and b; Supplementary Table 11). These independent predictors worked consistently between microarray and RNA-seq within GSE47792 (Fig. 7c).

### Evaluation of different patient stratification strategies

Finally, we evaluated our subgrouping method (named Hu_Subgroups) together with other reports. van Groningen and colleagues reported that neuroblastoma is composed of 2 super-enhancer-associated differentiation states: an ‘ADRN’ subgroup showing up-regulated genes involved in adrenergic differentiation and an ‘MES’ subgroup with higher expressions of mesenchymal markers^35^. To quantify these characteristics, we calculated the "ADRN" or "MES" scores of our subgroups based on a 369-gene "ADRN" signature or a 485-gene MES signature, respectively. We observed that subgroup 3 showed the highest "MES" scores and the lowest "ADRN" scores, consistent with our above findings; while subgroups 1 and 2 had the highest "ADRN" scores with the lowest "MES" scores in subgroup 2 (Supplementary Fig. 8a and b).

**Figure 8:**
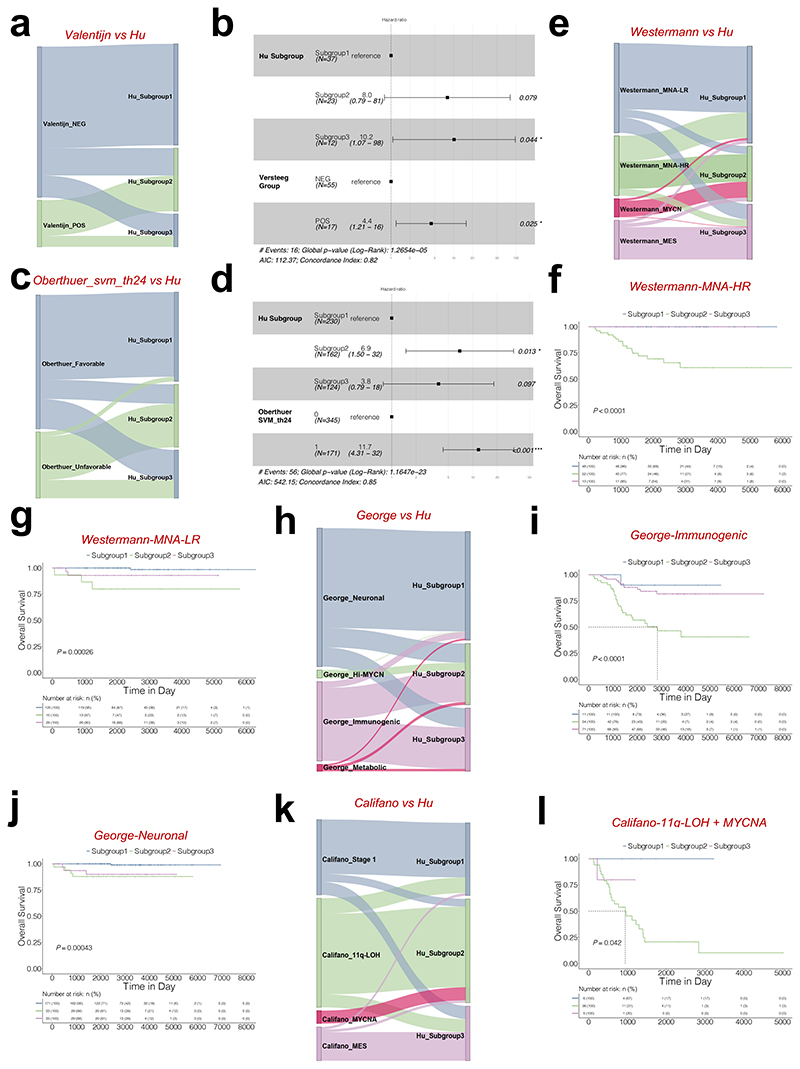
A systematic comparison of the subgroup classifier with previously published gene expression classifiers. (**a**) Prediction differences in GSE16476 using the subgrouping method from this report (named Hu) or Valentijn and colleagues (Valentijn). (**b**) Multivariate analysis of subgroup classification with Valentijn classification in *MYCN* non-amplified neuroblastomas. HR (hazard ratio), 95% CI (confidence interval), patient number (n), and *P* values are shown. (**c**) Prediction differences in E-MTAB-1781 using the subgrouping method from this report (named Hu) or Oberthuer and colleagues (Oberthuer's svm_th24). (**d**) Multivariate analysis of subgroup classification with Oberthuer's svm_th24 classification in *MYCN* non-amplified neuroblastomas. HR (hazard ratio), 95% CI (confidence interval), patient number (n), and *P* values are shown. (**e**) Prediction differences in GSE49711 using the subgrouping method from this report (named Hu) or Westermann and colleagues (Westermann). Kaplan-Meier plots showing the overall survival in Westermann_MNA-HR (**f**) or Westermann_MNA-LR (**g**) patients using the subgrouping method from this report. Numbers below are n (%). P values are indicated. (**h**) Prediction differences in GSE49711 using subgrouping method from this report (named Hu) or George and colleagues (George). Kaplan-Meier plots showing the overall survival in George_Immunogenic (**i**) or George_Neuronal (**j**) patients using the subgrouping method from this report. The numbers below are n (%). *P* values are indicated. (**k**) Prediction differences in GSE85047 using the subgrouping method from this report (named Hu) or Califano and colleagues (Califano). (**l**) Kaplan-Meier plots showing the overall survival in Califano_11q-LOH & MYCNA patients using the subgrouping method from this report. The numbers below are n (%). *P* values are indicated.

We also compared Hu_Subgroups with the Valentijn classification^19^. All subgroup 1 samples (n = 33) and two-thirds of subgroup 3 (n = 8) belonged to Valentijn's NEG group, while 13 out of 23 subgroup 2 samples were part of Valentijn's POS group (Fig. 8a; Supplementary Table 12). In addition, the multivariate analysis indicated that our subgroup 3 could be an independent variable after being adjusted by Valentijn's classifier (Fig. 8b).

Since 2006, Oberthuer and colleagues have been dedicated to constructing a molecular classification system capable of accurately categorizing patients into favourable and unfavourable groups, continually iterating over the following decade^36–39^. The most recent molecular predictors NB-th24 and NB-th44 were introduced in 2017^40^. A comparative analysis between our model and their two models reveals a strong consistency in the favourable and unfavourable outcomes of the respective groupings (Fig. 8c). Specifically, 218 out of 230 subgroup 1 samples and 77 out of 124 subgroup 3 samples were labelled as the favourable group based on SVM_th24. Conversely, more than half of the subgroup 2 samples were categorized as unfavourable (Supplementary Table 12). Similar results were identified in the SVM_th44 comparison (Supplementary Fig. 8c). Additionally, multivariate analysis to determine subgroup 2 could serve as an independent variable after adjusting for Oberthuer’s classifier (Fig. 8d; Supplementary Fig. 8d).

Recently, Westermann and colleagues reported 4 subgroups in neuroblastoma, including *MYCN*-amplified (MYCN), *MYCN* non-amplified high-risk (MNA-HR), *MYCN* non-amplified low-risk (MNA-LR) and mesenchymal (MES)^41^. With our method, patients within Westermann_MNA-HR can be further classified into 3 subtypes (Fig. 8e), showing different prognosis (Fig. 8f). This was also true for Westermann_MNA-LR (Fig. 8g). A majority of cases in Westermann_MES or MYCN belonged to subgroup 3 and 2, respectively (Fig. 8e).

George and colleagues classified 498 neuroblastoma samples into 4 distinct clusters based on RNA-seq profiles^42^. These clusters include the George_Hi-MYCN cluster, characterized by *MYCN* target genes; the George_neuronal cluster, predominantly composed of *MYCN* non-amplified tumours; the George_immunogenic cluster, enrichment of immune genes; and the George_metabolic cluster, encompassing the remaining samples. A substantial portion of the George_neuronal cluster and the George_immunogenic cluster fall into subgroups 1 and 3, respectively. Specifically, 13 out of 14 samples from the George_Hi-MYCN cluster are categorized to subgroup 2 (Fig. 8h). Notably, subgroups within the George_immunogenic cluster and George_neuronal cluster also demonstrate distinct survival outcomes (Fig. 8i and j).

Califano and colleagues classified high-risk neuroblastomas into 3 main subgroups (MYCN^Amp^, MYCNA), 11q-LOH (loss of heterozygosity), and mesenchymal (MES)^43^. In comparison, in the GSE85047 microarray, all cases of Califano_ MYCNA were classified in subgroup 2. Most cases in Califano_ MES or Stage1 belonged to subgroups 3 and 1 respectively (Fig. 8k). Interestingly, most cases in Califano_11q-LOH were classified in subgroup 2 (Fig. 8k), and subgroups within Califano_11q-LOH and MYCNA exhibit different survival results (Fig. 8l).

Together with other reports, our findings emphasised the extent of inner heterogeneity within the *MYCN* non-amplified population and the importance of patient stratification.

## Discussion

Neuroblastoma remains a challenge in the era of personalised therapy, largely due to inter- and intra-tumoral heterogeneity. Gene amplification in *MYCN* is the first genetic marker that indicates a highly invasive, advanced neuroblastoma, which has been observed in about 20% of primary and about 40% of high-risk neuroblastoma cases^44^. Despite the extensive study of *MYCN*-amplified neuroblastomas, there is a significant unmet clinical need in *MYCN* non-amplified neuroblastomas.

In this study, using tumour expression data and ConsensusClusterPlus, we demonstrate that *MYCN* non-amplified neuroblastomas are heterogeneous and can be further classified into 3 subgroups based on their transcriptional profiling. Within them, subgroup 2 has the worst prognosis and this group exhibits a "*MYCN*" signature that is potentially induced by the overexpression of AURKA. AURKA interacts with both N-MYC and SCF (Fbxw7) ubiquitin ligase, which ubiquitinates N-MYC for degradation. Consequently, overexpression of AURKA counteracts the degradation of N-MYC, leading to the growth of neuroblastoma cells^21,45^.

Subgroup 3 is accompanied by EMT and an "inflamed" phenotype, with high expression of genes related to IL2_STAT5 signaling, IL6 JAK STAT3 signaling, interferon-α activation, interferon-γ activation, and inflammatory response, consistent with the association between EMT and immune-related gene expression^46,47^. The findings were further confirmed by using CIBERSORTx deconvolution^25^ to quantify various immune populations based upon a *MYCN* non-amplified neuroblastoma scRNA-seq data^26^, showing increased percentages of fibroblasts, B cells, myeloid cells, T cells, and pDC (plasmacytoid dendritic cells).

The clinical implications of this subtype classification are significant, as each subtype demonstrates a unique prognosis and vulnerability to investigational therapies. For example, patients in subgroup 1 show the most favourable prognosis with a long-term survival rate above 85%, despite some of them being clinically classified as INSS stage IV or high risk. It might suggest that we should take a more careful and precise evaluation of some patients in reality after the consideration of all clinical information such as age, stage, risk status, or our stratification rather than making a decision based on a single parameter. With regard to therapy stratification, evidence showing significantly high MHC-I and CYT scores in subgroup 3 suggests that patients within this group may benefit from immunotherapy. Our analysis suggests that subgroup 3 is predicted to respond to anti-PD1 immunotherapy. The application of immunotherapy in neuroblastoma has started with treatments such as GD2 monoclonal antibody (dinutuximab) and Chimeric antigen receptor T cells (CAR-T) therapy^48,49^. Further studies, including in *vitro*, in *vivo*, and clinical validations, are required to investigate if patients within subgroup 3 can benefit from immunotherapy.

In addition, our study suggests that patients within subgroup 2 may benefit from AURKA inhibitors that can disrupt the interaction between AURKA and N-MYC. Indeed, AURKA inhibitors, MLN8054 and MLN8237 (Alisertib), are able to disrupt this interaction, leading to N-MYC degradation and subsequently cell death and differentiation in neuroblastoma cells^45,50^. MLN8237 (Alisertib) is currently under phase 2 clinical evaluation in neuroblastoma (NCT01154816).

With the establishment of independent predictors, *MYCN* non-amplified neuroblastomas were easily classified into one of the 3 subtypes, permitting a realistic scenario in which prospective subtyping is performed in a cohort, wherein patients are assigned to different therapeutics (*e*.*g*., subgroup 3 to immunotherapy, subgroup 2 to AURKA inhibitors) based on their subtype. If any one of these predictions demonstrated significant benefit, it would represent the first standard-of-care molecular biomarker selection for *MYCN* non-amplified neuroblastomas and a foundational step toward personalised therapy for this devastating disease.

## Methods

### Subtype identification

The study design is provided in Fig. 1a with a summary of datasets in the Supplementary Table S1. A detailed description of the approach and further characterisation of the subtypes by principal component analysis (PCA), ConsensusClusterPlus, single-sample Gene Set Enrichment Analysis (ssGSEA), weighted gene co-expression network analysis (WGCNA), and CIBERSORTx analysis is provided in the Supplementary Methods.

### Analysis of hazard ratio and overall survival

The univariate and multivariate Cox proportional hazards model assessed the hazard ratio of each parameter through the survminer (v0.4.9). We performed a log-rank test to compare Kaplan-Meier survival curves between each subgroup by survival (v3.2-10). Prediction error curves of each prognostic model were generated from pec (v2019.11.03)^51^.

### Analysis of clinically actionable genes and drug response

To investigate subgroup-specific druggable targets, we performed an integrative analysis to assess the associations between molecular features and the response to anticancer drugs in *MYCN* non-amplified neuroblastomas. A detailed description of the approach is provided in Supplementary Methods.

### Identification of independent predictors

To identify independent predictors for subgrouping, we applied a multi-cohort analysis pipeline via MetaIntegrator^30^ and validated with the machine learning classifier, support vector machine (SVM) (see details in Supplementary Methods).

### Tissue microarray (TMA) preparation and immunohistochemistry (IHC)

Separate a small part of the tissue specimen and shape it in a customized mold for chip production and fix it overnight in 4% paraformaldehyde (PFA). Tissue blocks were embedded in paraffin in a prepared array. Then the sample was sliced (5 μm) and adhered to a poly-L-lysine coated glass slide for immunohistochemical staining, which was performed as previously described^52,53^, using specific antibody against N-MYC (1:600 dilutions; Cell Signaling Technology 51705) and Aurora kinase A (1:200 dilutions; Abcam ab52973). Blindly, with no knowledge of the clinicopathological characteristics of the tumour, the immunoreactivity in tissue sections was observed under three microscopes at random and then evaluated by 3 pathologists. Differences in scoring were discussed until a consensus was reached. The tissue sections were then scored under an optical microscope according to the degree of staining (0 ∼ 3 points were negative staining, light yellow, light brown, dark brown) and the positive range (1 ∼ 4 points were 0 ∼ 25%, 26 ∼ 50%, 51 ∼ 75%, 76 ∼ 100%). Finally, samples were divided into a high-expression group and a low-expression group based on the median of the final staining score. All procedures adhered to the ethical standards set by the Clinical Committee of Xinhua Hospital, Shanghai Jiao Tong University School of Medicine (Approval No: XHEC-D-2016-037).

## Supplementary Material

Supplementary Materials

Table S1

Table S2

Table S3

Table S4

Table S5

Table S6

Table S7

Table S8

Table S9

Table S10

Table S11

Table S12

## Data Availability

Codes were implemented in R and have been deposited in GitHub: https://github.com/yz3n18/neuroblastoma.

## Data availability

All data supporting the findings of the current study are listed in Supplementary Materials.

## References

[1] Stiller CA, Parkin DM (1992). International variations in the incidence of neuroblastoma. Int J Cancer.

[2] Tsubota S, Kadomatsu K (2018). Origin and initiation mechanisms of neuroblastoma. Cell Tissue Res.

[3] London WB, Castleberry RP, Matthay KK, Look AT, Seeger RC, Shimada H (2005). Evidence for an age cutoff greater than 365 days for neuroblastoma risk group stratification in the Children's Oncology Group. J Clin Oncol.

[4] Song X, Huang C, Wang S, Yan L, Wang J, Li Y (2020). Neck management in patients with olfactory neuroblastoma. Oral Oncol.

[5] Boeva V, Louis-Brennetot C, Peltier A, Durand S, Pierre-Eugène C, Raynal V (2017). Heterogeneity of neuroblastoma cell identity defined by transcriptional circuitries. Nat Genet.

[6] London WB, Castel V, Monclair T, Ambros PF, Pearson AD, Cohn SL (2011). Clinical and biologic features predictive of survival after relapse of neuroblastoma: a report from the International Neuroblastoma Risk Group project. J Clin Oncol.

[7] Cohn SL, Pearson AD, London WB, Monclair T, Ambros PF, Brodeur GM (2009). The International Neuroblastoma Risk Group (INRG) classification system: an INRG Task Force report. J Clin Oncol.

[8] Bell E, Lunec J, Tweddle DA (2007). Cell cycle regulation targets of MYCN identified by gene expression microarrays. Cell Cycle.

[9] Kang JH, Rychahou PG, Ishola TA, Qiao J, Evers BM, Chung DH (2006). MYCN silencing induces differentiation and apoptosis in human neuroblastoma cells. Biochem Biophys Res Commun.

[10] Wakamatsu Y, Watanabe Y, Nakamura H, Kondoh H (1997). Regulation of the neural crest cell fate by N-myc: promotion of ventral migration and neuronal differentiation. Development.

[11] Meitar D, Crawford SE, Rademaker AW, Cohn SL (1996). Tumor angiogenesis correlates with metastatic disease, N-myc amplification, and poor outcome in human neuroblastoma. J Clin Oncol.

[12] Goodman LA, Liu BC, Thiele CJ, Schmidt ML, Cohn SL, Yamashiro JM (1997). Modulation of N-myc expression alters the invasiveness of neuroblastoma. Clin Exp Metastasis.

[13] Brodeur GM, Seeger RC, Schwab M, Varmus HE, Bishop JM (1984). Amplification of N-myc in untreated human neuroblastomas correlates with advanced disease stage. Science.

[14] Brodeur GM, Seeger RC, Schwab M, Varmus HE, Bishop JM (1985). Amplification of N-myc sequences in primary human neuroblastomas: correlation with advanced disease stage. Prog Clin Biol Res.

[15] Look AT, Hayes FA, Shuster JJ, Douglass EC, Castleberry RP, Bowman LC (1991). Clinical relevance of tumor cell ploidy and N-myc gene amplification in childhood neuroblastoma: a Pediatric Oncology Group study. J Clin Oncol.

[16] Colon NC, Chung DH (2011). Neuroblastoma. Adv Pediatr.

[17] Hoshida Y, Brunet JP, Tamayo P, Golub TR, Mesirov JP (2007). Subclass mapping: identifying common subtypes in independent disease data sets. PLoS One.

[18] Langfelder P, Horvath S (2008). WGCNA: an R package for weighted correlation network analysis. BMC Bioinformatics.

[19] Valentijn LJ, Koster J, Haneveld F, Aissa RA, van Sluis P, Broekmans ME (2012). Functional MYCN signature predicts outcome of neuroblastoma irrespective of MYCN amplification. Proc Natl Acad Sci U S A.

[20] Romain C, Paul P, Kim KW, Lee S, Qiao J, Chung DH (2014). Targeting Aurora kinase-A downregulates cell proliferation and angiogenesis in neuroblastoma. J Pediatr Surg.

[21] Otto T, Horn S, Brockmann M, Eilers U, Schüttrumpf L, Popov N (2009). Stabilization of N-Myc is a critical function of Aurora A in human neuroblastoma. Cancer Cell.

[22] Jin W, Zhang Y, Liu Z, Che Z, Gao M, Peng H (2021). Exploration of the molecular characteristics of the tumor-immune interaction and the development of an individualized immune prognostic signature for neuroblastoma. J Cell Physiol.

[23] Li Y, Jiang T, Zhou W, Li J, Li X, Wang Q (2020). Pan-cancer characterization of immune-related lncRNAs identifies potential oncogenic biomarkers. Nat Commun.

[24] Yoshihara K, Shahmoradgoli M, Martínez E, Vegesna R, Kim H, Torres-Garcia W (2013). Inferring tumour purity and stromal and immune cell admixture from expression data. Nat Commun.

[25] Newman AM, Liu CL, Green MR, Gentles AJ, Feng W, Xu Y (2015). Robust enumeration of cell subsets from tissue expression profiles. Nat Methods.

[26] Dong R, Yang R, Zhan Y, Lai HD, Ye CJ, Yao XY (2020). Single-Cell Characterization of Malignant Phenotypes and Developmental Trajectories of Adrenal Neuroblastoma. Cancer Cell.

[27] Roh W, Chen PL, Reuben A, Spencer CN, Prieto PA, Miller JP (2017). Integrated molecular analysis of tumor biopsies on sequential CTLA-4 and PD-1 blockade reveals markers of response and resistance. Sci Transl Med.

[28] Hugo W, Zaretsky JM, Sun L, Song C, Moreno BH, Hu-Lieskovan S (2016). Genomic and Transcriptomic Features of Response to Anti-PD-1 Therapy in Metastatic Melanoma. Cell.

[29] Su Y, Luo B, Lu Y, Wang D, Yan J, Zheng J (2022). Anlotinib Induces a T Cell-Inflamed Tumor Microenvironment by Facilitating Vessel Normalization and Enhances the Efficacy of PD-1 Checkpoint Blockade in Neuroblastoma. Clin Cancer Res.

[30] Haynes WA, Vallania F, Liu C, Bongen E, Tomczak A, Andres-Terrè M (2017). EMPOWERING MULTI-COHORT GENE EXPRESSION ANALYSIS TO INCREASE REPRODUCIBILITY. Pac Symp Biocomput.

[31] Wang C, Gong B, Bushel PR, Thierry-Mieg J, Thierry-Mieg D, Xu J (2014). The concordance between RNA-seq and microarray data depends on chemical treatment and transcript abundance. Nat Biotechnol.

[32] (2023). Therapeutically Applicable Research to Generate Effective Treatments-phs000467.

[33] Hartlieb SA, Sieverling L, Nadler-Holly M, Ziehm M, Toprak UH, Herrmann C (2021). Alternative lengthening of telomeres in childhood neuroblastoma from genome to proteome. Nat Commun.

[34] Hagemann S, Misiak D, Bell JL, Fuchs T, Lederer MI, Bley N (2023). IGF2BP1 induces neuroblastoma via a druggable feedforward loop with MYCN promoting 17q oncogene expression. Mol Cancer.

[35] van Groningen T, Koster J, Valentijn LJ, Zwijnenburg DA, Akogul N, Hasselt NE (2017). Neuroblastoma is composed of two super-enhancer-associated differentiation states. Nat Genet.

[36] Oberthuer A, Berthold F, Warnat P, Hero B, Kahlert Y, Spitz R (2006). Customized oligonucleotide microarray gene expression-based classification of neuroblastoma patients outperforms current clinical risk stratification. J Clin Oncol.

[37] Oberthuer A, Hero B, Berthold F, Juraeva D, Faldum A, Kahlert Y (2010). Prognostic impact of gene expression-based classification for neuroblastoma. J Clin Oncol.

[38] Garcia I, Mayol G, Ríos J, Domenech G, Cheung NK, Oberthuer A (2012). A three-gene expression signature model for risk stratification of patients with neuroblastoma. Clin Cancer Res.

[39] Oberthuer A, Juraeva D, Hero B, Volland R, Sterz C, Schmidt R (2015). Revised risk estimation and treatment stratification of low- and intermediate-risk neuroblastoma patients by integrating clinical and molecular prognostic markers. Clin Cancer Res.

[40] Rosswog C, Schmidt R, Oberthuer A, Juraeva D, Brors B, Engesser A (2017). Molecular Classification Substitutes for the Prognostic Variables Stage, Age, and MYCN Status in Neuroblastoma Risk Assessment. Neoplasia.

[41] Gartlgruber M, Sharma AK, Quintero A, Dreidax D, Jansky S, Park Y-G (2020). Super enhancers define regulatory subtypes and cell identity in neuroblastoma. Nature Cancer.

[42] Sengupta S, Das S, Crespo AC, Cornel AM, Patel AG, Mahadevan NR (2022). Mesenchymal and adrenergic cell lineage states in neuroblastoma possess distinct immunogenic phenotypes. Nat Cancer.

[43] Rajbhandari P, Lopez G, Capdevila C, Salvatori B, Yu J, Rodriguez-Barrueco R (2018). Cross-Cohort Analysis Identifies a TEAD4-MYCN Positive Feedback Loop as the Core Regulatory Element of High-Risk Neuroblastoma. Cancer Discov.

[44] Seeger RC, Brodeur GM, Sather H, Dalton A, Siegel SE, Wong KY (1985). Association of multiple copies of the N-myc oncogene with rapid progression of neuroblastomas. N Engl J Med.

[45] Brockmann M, Poon E, Berry T, Carstensen A, Deubzer HE, Rycak L (2013). Small molecule inhibitors of aurora-a induce proteasomal degradation of N-myc in childhood neuroblastoma. Cancer Cell.

[46] Terry S, Savagner P, Ortiz-Cuaran S, Mahjoubi L, Saintigny P, Thiery JP (2017). New insights into the role of EMT in tumor immune escape. Mol Oncol.

[47] Dongre A, Rashidian M, Reinhardt F, Bagnato A, Keckesova Z, Ploegh HL (2017). Epithelial-to-Mesenchymal Transition Contributes to Immunosuppression in Breast Carcinomas. Cancer Res.

[48] Mujoo K, Cheresh DA, Yang HM, Reisfeld RA (1987). Disialoganglioside GD2 on human neuroblastoma cells: target antigen for monoclonal antibody-mediated cytolysis and suppression of tumor growth. Cancer Res.

[49] Richards RM, Sotillo E, Majzner RG (2018). CAR T Cell Therapy for Neuroblastoma. Front Immunol.

[50] Yang Y, Ding L, Zhou Q, Fen L, Cao Y, Sun J (2020). Silencing of AURKA augments the antitumor efficacy of the AURKA inhibitor MLN8237 on neuroblastoma cells. Cancer Cell Int.

[51] Mogensen UB, Ishwaran H, Gerds TA (2012). Evaluating Random Forests for Survival Analysis using Prediction Error Curves. J Stat Softw.

[52] Wang Y, Chen K, Cai Y, Cai Y, Yuan X, Wang L (2017). Annexin A2 could enhance multidrug resistance by regulating NF-κB signaling pathway in pediatric neuroblastoma. J Exp Clin Cancer Res.

[53] Gu Y, Lv F, Xue M, Chen K, Cheng C, Ding X (2018). The deubiquitinating enzyme UCHL1 is a favorable prognostic marker in neuroblastoma as it promotes neuronal differentiation. J Exp Clin Cancer Res.

