# Supplementary material for: Identification of MYCN non-amplified neuroblastoma subgroups points towards molecular signatures for precision prognosis and therapy stratification
Source: Br J Cancer. Author manuscript; Available in PMC 2024 May 27. (PMC7616008; doi:10.1038/s41416-024-02666-y)
Supplement: Supplementary Materials [file EMS194837-supplement-Supplementary_Materials.pdf]

Identification of *MYCN* non-amplified neuroblastoma subgroups points towards molecular signatures for precision prognosis and therapy stratification

Supplementary Materials

Table of contents

Supplementary Methods ... **- 3 -**
1. Data Collection ... - 3 -
2. Data Preparation ... - 3 -
3. Quality Control ... - 3 -
4. Data Normalisation ... - 4 -
5. Consensus Clustering ... - 4 -
6. Clinical Data Merging ... - 4 -
7. Defining the Differentially Expressed Genes (DEGs) and Pathway Analysis ... - 5 -
8. Weighted Gene Co-expression Network Analysis (WGCNA) and Protein Protein Interaction (PPI) Analysis ... - 5 -
9. Clinical Characterisation of Subtypes ... - 5 -
10. SubMap Analysis ... - 6 -
11. Single Cell RNA-seq (scRNA-seq) Analysis ... - 6 -
12. CIBERSORTx Analysis ... - 6 -
13. Identification of Independent Predictors ... - 6 -
References ... **8**
Supplementary Figures ... **10**
Supplementary Figure 1. Identification of duplicate samples across different datasets. ... 10
Supplementary Figure 2. Identification of datasets with inconsistent *MYCN* signatures across different datasets. ... 11
Supplementary Figure 3. Characterisation of molecular subtypes in *MYCN* non-amplified neuroblastomas. ... 12
Supplementary Figure 4. Clinical characterisation of subtypes within *MYCN* non-amplified neuroblastomas identifies key distinguishing features. ... 13
Supplementary Figure 5. Defining molecular features of 3 subtypes in *MYCN* non-amplified neuroblastomas. ... 14
Supplementary Figure 6. Subgroup 2 shows a "*MYCN*" signature, potentially induced by Aurora Kinase A (AURKA) overexpression. ... 15
Supplementary Figure 7. Subgroup 3 is accompanied by an "inflamed" gene signature. ... 16
Supplementary Figure 8. Evaluation of different patient stratification strategies within *MYCN* non-amplified neuroblastomas ... 17
Supplementary Tables ... **18**
Table S1. List of datasets and samples collected for meta-analysis. ... 18
Table S2. List of top 50% variable genes for consensus clustering ... 18
Table S3. Univariate and multivariate regression analysis in *MYCN* non-amplified neuroblastomas. ... 18
Table S4. DEGs (differentially expressed genes) in subgroups. ... 18

Table S5. GSEA (gene set enrichment analysis) in subgroups. 18
Table S6. WGCNA (weighted gene co-expression network analysis) in subgroups. 18
Table S7. List of genes in PPI (protein–protein interaction) network analysis. 18
Table S8. IHC result from Xinhua hospital. 18
Table S9. List of 46 immune-related gene sets. 18
Table S10. List of predict genes and rules. 18
Table S11. Prediction results of multiple cohorts. 18
Table S12. Classification differences between different stratification strategies. 18

Supplementary Methods

1. Data Collection

We searched the keywords "(*MYCN*) OR (*MYC*) OR (*MYCN* amplification) AND (Neuroblastoma) AND (Homo sapiens)" and publication dates before 21/02/2022 in National Centre for Biotechnology Information (NCBI) Gene Expression Omnibus (GEO) and European Bioinformatics Institute (EBI) ArrayExpress (AE). Three hundred and eighty experiments were identified in the initial screening. Then, we included only datasets that are neuroblastoma samples and contain *MYCN* status information with at least 2 biological replicates. Datasets generated on platforms other than Illumina, Affymetrix or Agilent or detected genes less than 10,000 were excluded to avoid the technical mismatch between different platforms (Fig. 1a).

2. Data Preparation

Raw microarray files were downloaded and imported into the R environment (v4.0.2). The normalisation of raw data depended on the generated platform. Affymetrix datasets were performed by the rma function in affy (v1.66.0)[1] or oligo(v3.11)[2] packages. Agilent microarrays were normalised using the normalise BetweenArrays function. Illumina datasets were standardised by neqc function in limma (v3.44.3)[3]. Microarray probe IDs were mapped to gene symbol according to the GPL annotation files provided in NCBI. Probes mapped to multiple gene symbols were removed and genes mapped to multiple probe IDs were summarised by calculating the mean.

3. Quality Control

Before proceeding with further analysis, we systematically evaluated and ruled out the possibility of patient duplicates. Overall, there are 3 types of duplicate samples being deposited into different public databases. The first one is that datasets had been deposited to 2 different data repositories. For example, E-GEOD-45547 (ArrayExpress) and GSE45547 (GEO) contain identical neuroblastoma patients. Of course, the link information on the webpage can assist in avoiding the inclusion of duplicate samples during data collection. The second type includes subseries datasets belonging to 1 superseries. For example, the superseires GSE16254 is composed of 163 samples and 5 subseries, where GSE16476 includes 88 identical microarray data. In this case, duplicated samples could be directly filtered based on GSMID. The last and most challenging situation to identify involves duplicated samples in different data repositories or different subseries without being labelled information or using different GSMID.

To exclude these duplicates, we have implemented the following steps:

1). Download the raw microarray file of all samples.

2). Since GSE19274 was the only dataset generated by Illumina, no duplicates were identified. For Agilent-generated file, "Scan_OriginalGUID & gRatioSig2BkgeQC_NegCtrl" from raw file would be used as unique Sample ID. For files generated Affymetrix, md5 value of each raw file would be calculated and used as unique Sample ID. According to Supplementary Fig. 1a-c, we identified 679 duplicate samples out of 761 samples across the following datasets GSE79910, GSE73517, GSE120572, GSE49710, GSE45547, E-MTAB-8248, E-MTAB-179, E-MTAB-1781. All samples from GSE21713 and E-MEXP-3517 matched the samples in GSE85047. Similarly, all samples from GSE14880 had been deposited into GSE12460. Samples with same "Scan_OriginalGUID & gRatioSig2BkgeQC_NegCtrl" or md5 value were compiled into

Supplementary Table 1 and each value would only keep one sample for subsequent analysis. In summary, 1,620 samples passed the selection criteria.

3). To ensure reliability, we filtered out low-quality datasets by performing leave-one-out-cross validation. 17 out of 18 datasets were training data for optimising the *MYCN* amplification signature and evaluating the prediction performance of this signature on the one left. Two datasets (GSE73537 and GSE53371) were filtered out due to the low area under receiver operating characteristic (ROC) curve (AUROC) score (Supplementary Fig. 2). The generation of a *MYCN* amplification signature and the validation of this signature on the dataset left were run through MetaIntegrator package (v2.1.3)[4] with FDRThresh = 0.05 and effectSizeThresh = 1.3. After filtering, 1,566 samples were analysed in further analysis.

4). To ensure there were no duplicates among the 1,566 samples, pairwise correlation coefficients were computed for all samples to globally assess the possibility of duplicate patients. A correlation coefficient value of 1 would indicate duplicate samples. After excluding the diagonal values in the correlation matrix, the correlation coefficient values ranged from 0.2696574 to 0.9962434 (refer to Supplementary Fig. 1d; Supplementary Table 1). The highest correlation coefficient value was observed between 2 samples exhibiting distinct clinical features within the same dataset (GSE16237_GSM408944 and GSE16237_GSM408936).

Through the aforementioned methods, we are confident to conclude that the possibility of patient duplicates has been eliminated.

4. Data Normalisation

After performing the individual normalization method of each raw microarray data and removing low-quality datasets, qualified datasets (16 in total) were merged into one dataset using gene symbols as references. We used the ComBat[5] empiric-Bayes batch correction algorithm to run an overall normalization of all data sets, thereby eliminating the technical batch effect and ensuring all samples were comparable across different datasets (Supplementary Fig. 3a). Principal component analysis (PCA) was applied to visualise all samples in lower dimensional space by factoextra[6] (Supplementary Fig. 3b). The merged dataset was randomly split into a training cohort (n = 878) and a testing cohort (n = 375) in a 7:3 ratio using the caret package[7].

5. Consensus Clustering

Median absolute deviations were calculated for each gene and the top 50% most variant genes (n = 5,792) were extracted for consensus clustering (ConsensusClusterPlus, v1.52.0)[8]. The consensus was performed using K-means with Euclidean distance, 80% item resampling (pItem), 100% gene resampling (pFeature) and 10,000 iterations to generate the robust consensus clusters. The delta area plot and the ClusterConsensus score suggested a matrix with k = 3 displayed the highest stability within clusters and clearest cut among clusters (Supplementary Fig. 3c).

6. Clinical Data Merging

Although duplicate samples from different databases share the same scanID or md5 value, different collection or deposit processes may result in missing or different clinical data for some duplicated samples. For instances where clinical data is missing, such as the absence of gene mutation information for genes like 1p, 11q, and 17q in the GSE49710 database, this kind of information can be complemented by data from the GSE73517 database.

Therefore, by integrating clinical information from different databases, a more comprehensive clinical profile for each sample can be achieved. In cases of conflicting clinical

data, a majority rule approach is initially applied. For example, samples GSE73517_GSM1897056, EMTAB1781_252038210106_1_4 and GSE49710_GSM1205330 share the same scanID: 1fb161ea-3708-415a-a2f8-4d5c0847d993_1229.52. However, while GSE73517_GSM1897056 and EMTAB1781_252038210106_1_4 have a 'high' risk status, GSE49710_GSM1205330 reports a 'low' risk status. Therefore, the final risk status is determined as 'high'. In cases of conflicting survival time and survival status, the longest survival time among samples with a survival event is selected as the final result. For example, the sample EMTAB179_US22502540_252038210106_1_4, with the same scanID: 1fb161ea-3708-415a-a2f8-4d5c0847d993_1229.52, has a survival time of 2502 days and no survival event. GSE49710_GSM1205330 has a longer survival time of 3935 days with no survival event. Although EMTAB1781_252038210106_1_4 has the longest survival time of 3958 days, the absence of a corresponding survival event information precludes its use in survival analysis. Consequently, the final survival time used is 3,935 days with no survival event.

7. Defining the Differentially Expressed Genes (DEGs) and Pathway Analysis

The limma package was used to compare each subgroup to other subgroups. Genes with an absolute log2 fold change (FC) bigger than 1 and a false discovery rate (FDR) p value less than 0.05 adjusted by using Benjamini–Hochberg (BH) method (or q-value) were considered as differentially expressed genes (DEGs). Pathway enrichment analysis were generated through Metascape website (http://metascape.org). Parameters of Metascape were set as "5 minimum overlapping genes, $p < 0.05$ and 1.5 minimum enrichment factor"[9]. GSEA (gene set enrichment analysis) was performed using GSEA software (the Broad Institute platform, v4.0.3)[10] with the default settings and 1,000 gene set permutations. Single-sample GSEA (ssGSEA) scores of pathways were calculated by using ssGSEA in the GSVA (v1.36.2) package[11]. "*MYCN*", "ADRN (adrenergic)" and "MES (mesenchymal)" signatures were collected from the previous reports[12,13]. The MHC (major histocompatibility complex) score was calculated as the mean expression of HLA-A, HLA-B, HLA-C, TAP1, TAP2, NLRC5, PSMB9, PSMB8, and B2M[14]. The CYT (cytolytic activity) score was calculated as the geometric mean of GZMA and PRF1[15].

8. Weighted Gene Co-expression Network Analysis (WGCNA) and Protein Protein Interaction (PPI) Analysis

WGCNA[16] of the top 50% most variant genes (n = 5,792) was used to discover sets of genes with similar correlation patterns among subgroups. To choose the parameter (soft threshold) of the power adjacency function, we used the scale-free topology (SFT) criterion. We selected the power value as 6 because the model-fit saturation was above 0.8. The identification of modules was performed using cutreeDynamic function with the signed hybrid method, deepSplit = 2 and minClusterSize = 100. Next, automatic merging was performed using mergeCloseModules function with a cutHeight of ≤ 0.25, which means a Pearson correlation between module eigengenes of ≥ 0.75. PPI in each module was demonstrated by STRING (v11.0)[17] with high confidence score > 0.900 interaction. Visualisation of network was performed by gephi[18]. *MYCN* score associated genes were determined by two criteria: 1) up-expressed DEGs in subgroup 2 samples and 2) the Pearson correlation coefficient with the *MYCN* score is greater than 0.3 and a false discovery rate (FDR) p value less than 0.05.

9. Clinical Characterisation of Subtypes

The univariate and multivariate Cox proportional hazards model assessed the hazard ratio of each parameter through the survminer (v0.4.9)[19]. We performed log-rank test to compare Kaplan-Meier survival curves between each subgroup by survival (v3.2-10)[20]. Prediction error curves of each prognostic model were generated from pec (v2019.11.03)[21].

10. SubMap Analysis

To compare the subgroups across independent neuroblastoma cohorts, melanoma datasets (GSE78220)[22,23]and anlotinib dataset[24], we applied an unsupervised subclass mapping (SubMap) analysis from GenePattern module (https://www.genepattern.org/modules) to evaluate similarity of subgroups[25]. Gene used were the intersection of genes between two correspondent datasets. Module parameters were setting as default and a Bonferroni adjusted p value less than 0.05 was considered as the significant cut-off.

11. Single Cell RNA-seq (scRNA-seq) Analysis

A scRNA-seq dataset of 3 *MYCN* non-amplified neuroblastoma samples was downloaded from GEO (GSE137804)[26]. The processed gene expression matrix was imported into R and analysed by Seurat Packages[27]. We first removed low-quality cells with detected genes less than 500 and those with more than 10% genes from the mitochondrial genome. The filtered gene expression matrix was normalised by SCTransform function. Variable features across 3 sample were found using SelectIntegrationFeatures function and then identified anchors using the FindIntegrationAnchors function, followed by the IntegrateData function to integrate these 3 samples. The integrated data were visualised using Uniform Manifold Approximation and Projection (UMAP) with RunUMAP function. The annotation of each cell type in the integrated dataset was performed using established signatures from a previous report.

12. CIBERSORTx Analysis

A signature matrix of each cell type in *MYCN* non-amplified neuroblastoma samples was constructed by a deconvolutional tool CIBERSORTx[28]. Cell fractions in each partition from the train cohort as well as those from the test cohort were imputed using Cell Fraction analysis module with B-mode and other default parameters over 1,000 permutations[29].

13. Identification of Independent Predictors

Considering that the transcriptomic data is derived from different data platforms, such as Agilent/Affymetrix/Illumina and RNA-seq, distinct standardisation methods are applied to ensure comparability of gene expression levels across samples. However, such processing is heavily influenced by samples within the same cohort, making it challenging to reach consistent conclusions across independent datasets[30]. In contrast, predictors based on gene rules (also referred to as single sample predictors) are considered to mitigate the impact of gene standardisation, while being less dependent on the source of the data platform. Gene rules operate on the principle that if the expression level of gene A is greater than that of gene B, the sample is assigned to Class X; otherwise, it is assigned to Class Y. K-Top-Scoring Pairs (kTSP) has been demonstrated to perform well in binary classification scenarios[31-33]. However, as our grouping involves a multi-class problem, we have opted for the use of the multi-class predictor, multiclassPairs[34].

Raw microarray files of 16 datasets were imported into the R environment again. However, no standardisation was applied. Affymetrix were performed by threestep() with normalize=F; Agilent data were imported by read.maimages() with other.columns='gIsWellAboveBG' or 'gProcessedSignal' according to raw data files; Illumina data were read by read.ilmn() with other.columns='Detection'. Microarray probe IDs were mapped to gene symbol according to the GPL annotation files provided in NCBI. Probes mapped to multiple gene symbols were removed and genes mapped to multiple probe IDs were summarised by calculating the mean.
Subsequently, the non-standardised expression matrices were integrated into a merged expression matrix, which was then divided based on train/test grouping. The train data were utilised for training single-sample predictors, while the test data were employed for evaluating model performance. During the model construction, 'one-vs-one gene' was selected because it

would give more weight to small classes. A total of 928 rules derived from a set of 432 genes were constructed during the training. Subsequently, these 928 rules were employed to train the final model constructed by a random forest with 100,000 trees. The final RF model got 93.17%, 94.2% and 91.9% balanced accuracy in the training data and 86.71%, 84.72% and 80.85% balanced accuracy in the testing data.

References

1 Gautier, L., Cope, L., Bolstad, B. M. & Irizarry, R. A. affy--analysis of Affymetrix GeneChip data at the probe level. *Bioinformatics* **20**, 307-315 (2004).

2 Carvalho, B. S. & Irizarry, R. A. A framework for oligonucleotide microarray preprocessing. *Bioinformatics* **26**, 2363-2367 (2010).

3 Ritchie, M. E., Phipson, B., Wu, D., Hu, Y., Law, C. W., Shi, W. *et al.* limma powers differential expression analyses for RNA-sequencing and microarray studies. *Nucleic Acids Res* **43**, e47 (2015).

4 Haynes, W. A., Vallania, F., Liu, C., Bongen, E., Tomczak, A., Andres-Terrè, M. *et al.* EMPOWERING MULTI-COHORT GENE EXPRESSION ANALYSIS TO INCREASE REPRODUCIBILITY. *Pac Symp Biocomput* **22**, 144-153 (2017).

5 Leek, J. T., Johnson, W. E., Parker, H. S., Jaffe, A. E. & Storey, J. D. The sva package for removing batch effects and other unwanted variation in high-throughput experiments. *Bioinformatics* **28**, 882-883 (2012).

6 A. Kassambara & Mundt, F. factoextra: Extract and Visualize the Results of Multivariate Data Analyses. *R Package Version 1.0.7.* 10.4236/ojs.2015.54031 (2020).

7 Kuhn, M. caret: Classification and Regression Training. R package version 6.0-86. (2020).

8 Wilkerson, M. D. & Hayes, D. N. ConsensusClusterPlus: a class discovery tool with confidence assessments and item tracking. *Bioinformatics* **26**, 1572-1573 (2010).

9 Zhou, Y., Zhou, B., Pache, L., Chang, M., Khodabakhshi, A. H., Tanaseichuk, O. *et al.* Metascape provides a biologist-oriented resource for the analysis of systems-level datasets. *Nat Commun* **10**, 1523 (2019).

10 Subramanian, A., Tamayo, P., Mootha, V. K., Mukherjee, S., Ebert, B. L., Gillette, M. A. *et al.* Gene set enrichment analysis: a knowledge-based approach for interpreting genome-wide expression profiles. *Proc Natl Acad Sci U S A* **102**, 15545-15550 (2005).

11 Hänzelmann, S., Castelo, R. & Guinney, J. GSVA: gene set variation analysis for microarray and RNA-seq data. *BMC Bioinformatics* **14**, 7 (2013).

12 Valentijn, L. J., Koster, J., Haneveld, F., Aissa, R. A., van Sluis, P., Broekmans, M. E. *et al.* Functional MYCN signature predicts outcome of neuroblastoma irrespective of MYCN amplification. *Proc Natl Acad Sci U S A* **109**, 19190-19195 (2012).

13 van Groningen, T., Koster, J., Valentijn, L. J., Zwijnenburg, D. A., Akogul, N., Hasselt, N. E. *et al.* Neuroblastoma is composed of two super-enhancer-associated differentiation states. *Nat Genet* **49**, 1261-1266 (2017).

14 Lauss, M., Donia, M., Harbst, K., Andersen, R., Mitra, S., Rosengren, F. *et al.* Mutational and putative neoantigen load predict clinical benefit of adoptive T cell therapy in melanoma. *Nat Commun* **8**, 1738 (2017).

15 Rooney, M. S., Shukla, S. A., Wu, C. J., Getz, G. & Hacohen, N. Molecular and genetic properties of tumors associated with local immune cytolytic activity. *Cell* **160**, 48-61 (2015).

16 Langfelder, P. & Horvath, S. WGCNA: an R package for weighted correlation network analysis. *BMC Bioinformatics* **9**, 559 (2008).

17 Szklarczyk, D., Gable, A. L., Lyon, D., Junge, A., Wyder, S., Huerta-Cepas, J. *et al.* STRING v11: protein-protein association networks with increased coverage, supporting functional discovery in genome-wide experimental datasets. *Nucleic Acids Res* **47**, D607-d613 (2019).

18 Mathieu Bastian, S. H., Mathieu Jacomy. Gephi: an open source software for exploring and manipulating networks. International AAAI Conference on Weblogs and Social Media. (2009).

19 Kassambara A, K. M. survminer: Drawing Survival Curves using 'ggplot2'. R package version 0.4.9. (2019).

20 Therneau, T. A Package for Survival Analysis in S. R package version 3.2-10. (2015).

21 Mogensen, U. B., Ishwaran, H. & Gerds, T. A. Evaluating Random Forests for Survival Analysis using Prediction Error Curves. *J Stat Softw* **50**, 1-23 (2012).

22 Roh, W., Chen, P. L., Reuben, A., Spencer, C. N., Prieto, P. A., Miller, J. P. *et al.* Integrated molecular analysis of tumor biopsies on sequential CTLA-4 and PD-1 blockade reveals markers of response and resistance. *Sci Transl Med* **9** (2017).

23 Hugo, W., Zaretsky, J. M., Sun, L., Song, C., Moreno, B. H., Hu-Lieskovan, S. *et al.* Genomic and Transcriptomic Features of Response to Anti-PD-1 Therapy in Metastatic Melanoma. *Cell* **168**, 542 (2017).

24 Su, Y., Luo, B., Lu, Y., Wang, D., Yan, J., Zheng, J. *et al.* Anlotinib Induces a T Cell-Inflamed Tumor Microenvironment by Facilitating Vessel Normalization and Enhances the Efficacy of PD-1 Checkpoint Blockade in Neuroblastoma. *Clin Cancer Res* **28**, 793-809 (2022).

25 Reich, M., Liefeld, T., Gould, J., Lerner, J., Tamayo, P. & Mesirov, J. P. GenePattern 2.0. *Nat Genet* **38**, 500-501 (2006).

26 Dong, R., Yang, R., Zhan, Y., Lai, H. D., Ye, C. J., Yao, X. Y. *et al.* Single-Cell Characterization of Malignant Phenotypes and Developmental Trajectories of Adrenal Neuroblastoma. *Cancer Cell* **38**, 716-733.e716 (2020).

27 Stuart, T., Butler, A., Hoffman, P., Hafemeister, C., Papalexi, E., Mauck, W. M., 3rd *et al.* Comprehensive Integration of Single-Cell Data. *Cell* **177**, 1888-1902.e1821 (2019).

28 Newman, A. M., Steen, C. B., Liu, C. L., Gentles, A. J., Chaudhuri, A. A., Scherer, F. *et al.* Determining cell type abundance and expression from bulk tissues with digital cytometry. *Nat Biotechnol* **37**, 773-782 (2019).

29 Le, T., Aronow, R. A., Kirshtein, A. & Shahriyari, L. A review of digital cytometry methods: estimating the relative abundance of cell types in a bulk of cells. *Brief Bioinform* **22** (2021).

30 Cirenajwis, H., Lauss, M., Planck, M., Vallon-Christersson, J. & Staaf, J. Performance of gene expression-based single sample predictors for assessment of clinicopathological subgroups and molecular subtypes in cancers: a case comparison study in non-small cell lung cancer. *Brief Bioinform* **21**, 729-740 (2020).

31 Tan, A. C., Naiman, D. Q., Xu, L., Winslow, R. L. & Geman, D. Simple decision rules for classifying human cancers from gene expression profiles. *Bioinformatics* **21**, 3896-3904 (2005).

32 Paquet, E. R. & Hallett, M. T. Absolute assignment of breast cancer intrinsic molecular subtype. *J Natl Cancer Inst* **107**, 357 (2015).

33 Xu, L., Tan, A. C., Naiman, D. Q., Geman, D. & Winslow, R. L. Robust prostate cancer marker genes emerge from direct integration of inter-study microarray data. *Bioinformatics* **21**, 3905-3911 (2005).

34 Marzouka, N. A. & Eriksson, P. multiclassPairs: an R package to train multiclass pair-based classifier. *Bioinformatics* **37**, 3043-3044 (2021).

35 Sengupta, S., Das, S., Crespo, A. C., Cornel, A. M., Patel, A. G., Mahadevan, N. R. *et al.* Mesenchymal and adrenergic cell lineage states in neuroblastoma possess distinct immunogenic phenotypes. *Nat Cancer* **3**, 1228-1246 (2022).

Supplementary Figures

Supplementary Figure 1. Identification of duplicate samples across multiple datasets. UpSet plot representations of duplicate samples across numerous datasets. Duplicate samples generated from Agilent_GPL16876 (**a**), Affymetrix_GPL570 (**b**) and Affymetrix_GPL5175 (**c**). (**d**) Violin plot of pairwise correlation coefficient values of 1,566 unique samples.

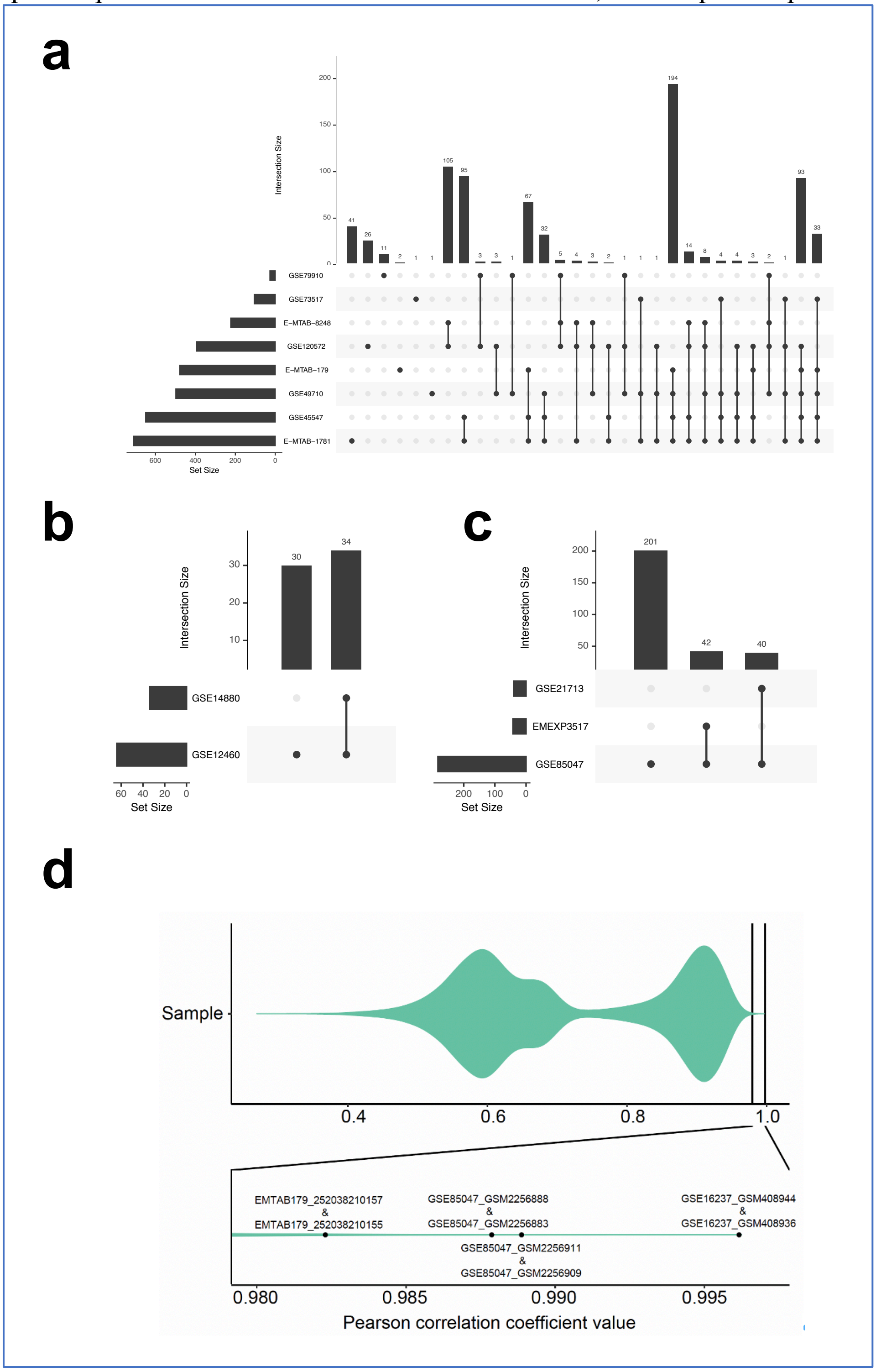

Supplementary Figure 2. Identification of datasets with inconsistent *MYCN* signatures across different datasets. AUROC (area under the receiver-operating characteristic curve) analysis comparing the performance of *MYCN* signatures trained from 17 datasets in the remaining one dataset. The dataset with AUROC scores less than 0.5 would be excluded.

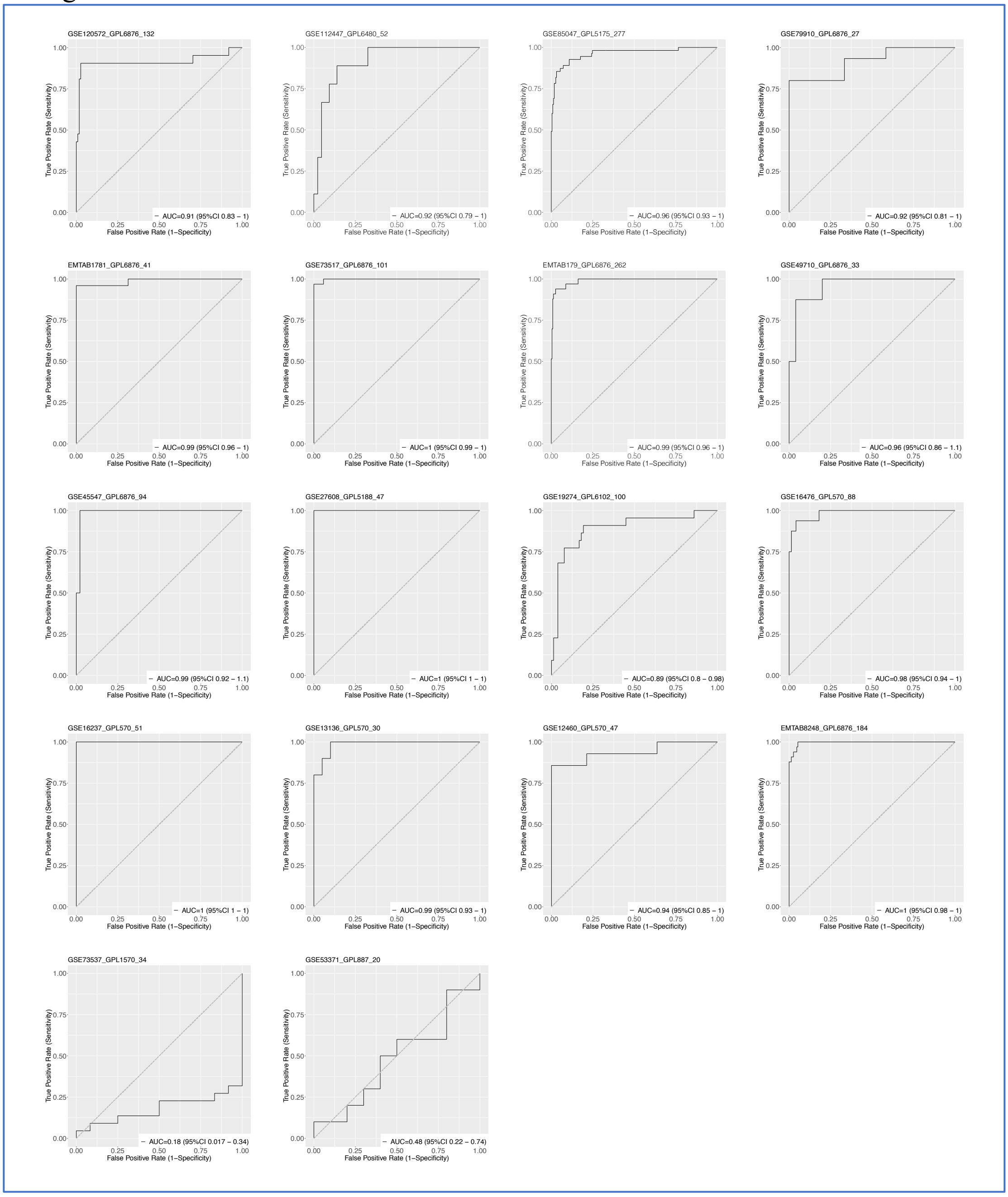

Supplementary Figure 3. Characterisation of molecular subtypes in *MYCN* non-amplified neuroblastomas. (**a**) Boxplot of merging neuroblastoma tumour sample from 18 datasets before and after removing batch effects. (**b**) Principal component analysis (PCA) of neuroblastoma patients after removing batch effect showed patients clustered according to *MYCN*-amplified status while there were overlapping areas between the two groups. (**c**) Relative area changes on the cumulative distribution function and cluster-consensus value of the train or test cohort.

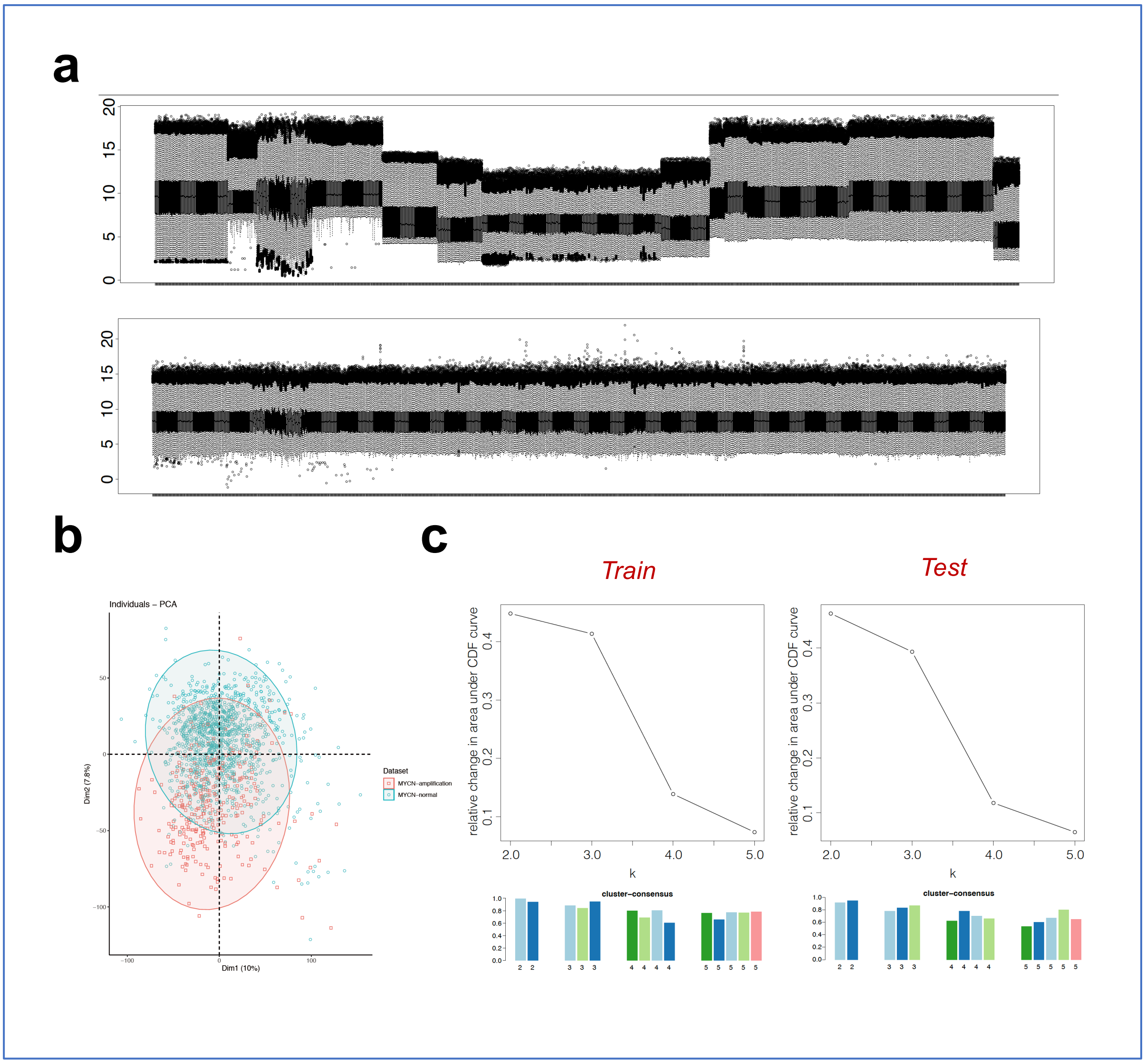

Supplementary Figure 4. Clinical characterisation of subtypes within *MYCN* non-amplified neuroblastomas identifies key distinguishing features. (**a**) SubMap analysis of 3 subgroups between the train and test cohort. Bonferroni adjusted *P*-values were indicated. (**b**) Graphs showing the frequency (%) of each molecular subtype in different International Neuroblastoma Staging System (INSS) stages or risk status in the train plus test cohort. *P* values are indicated. (**c**) Kaplan-Meier plots showing the overall survival in each molecular subtype or *MYCN*-amplification (*MYCN*-AMP) in the train plus test cohort. Numbers below are n (%). *P* values are indicated. (**d**) Multivariate analysis of subgroup classification with clinical parameters age, gender, INSS stage, risk status, *MYCN* status and 1p status in neuroblastomas. (**e** and **f**) Multivariate analysis of subgroup classification with 1p/11q/17q status in neuroblastomas. HR (hazard ratio), 95% CI (confidence interval), patient number (n) and p values are shown. (**g**) Prediction error curves (indicating mean squared error in predicting survival status) are calculated for the subgroup (red) and risk status (green).

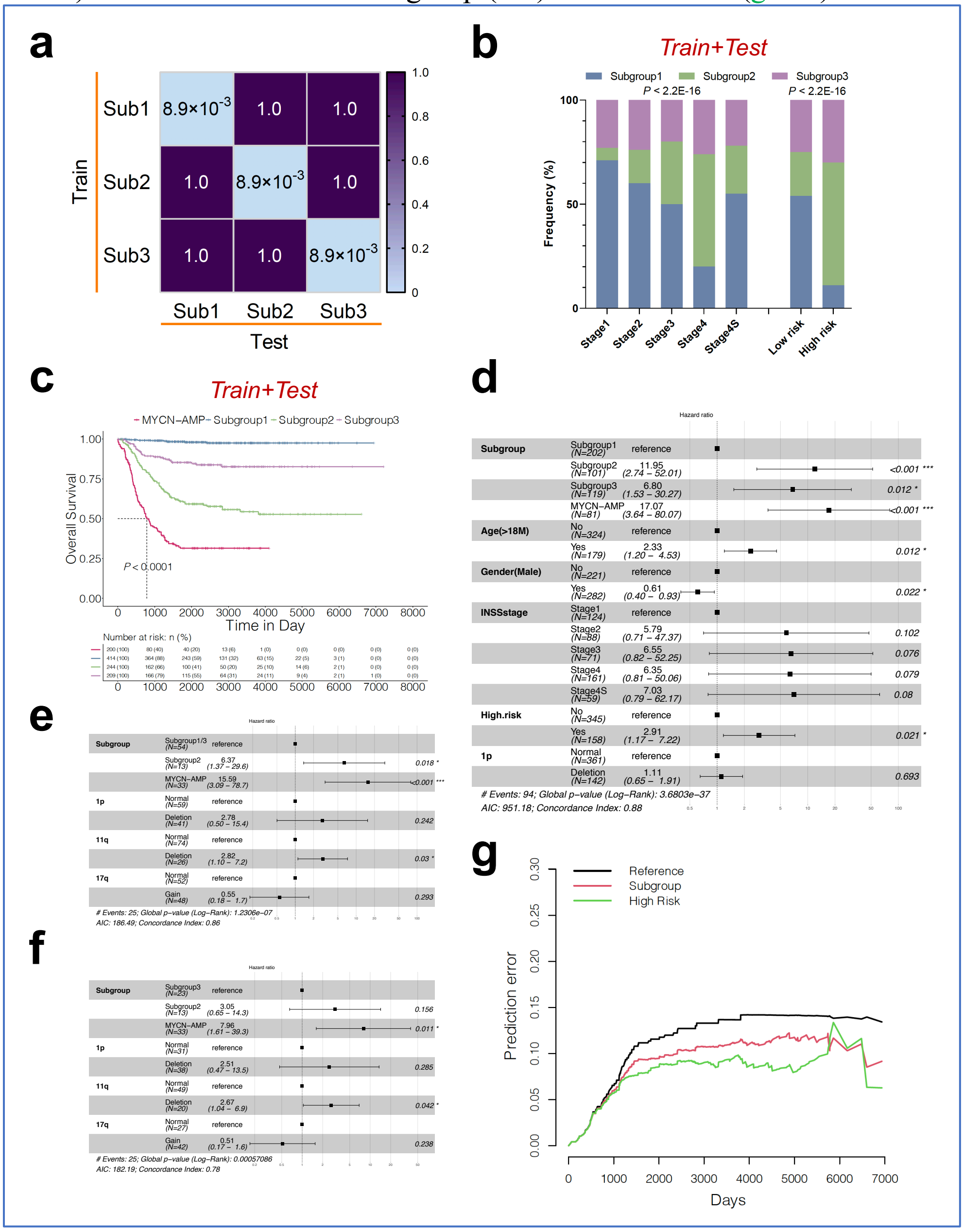

Supplementary Figure 5. Defining molecular features of 3 subtypes in *MYCN* non-amplified neuroblastomas. The correlation coefficients of WGCNA (weighted gene co-expression network analysis) modules and subgroups (red indicates positive correlated and blue negative correlated).

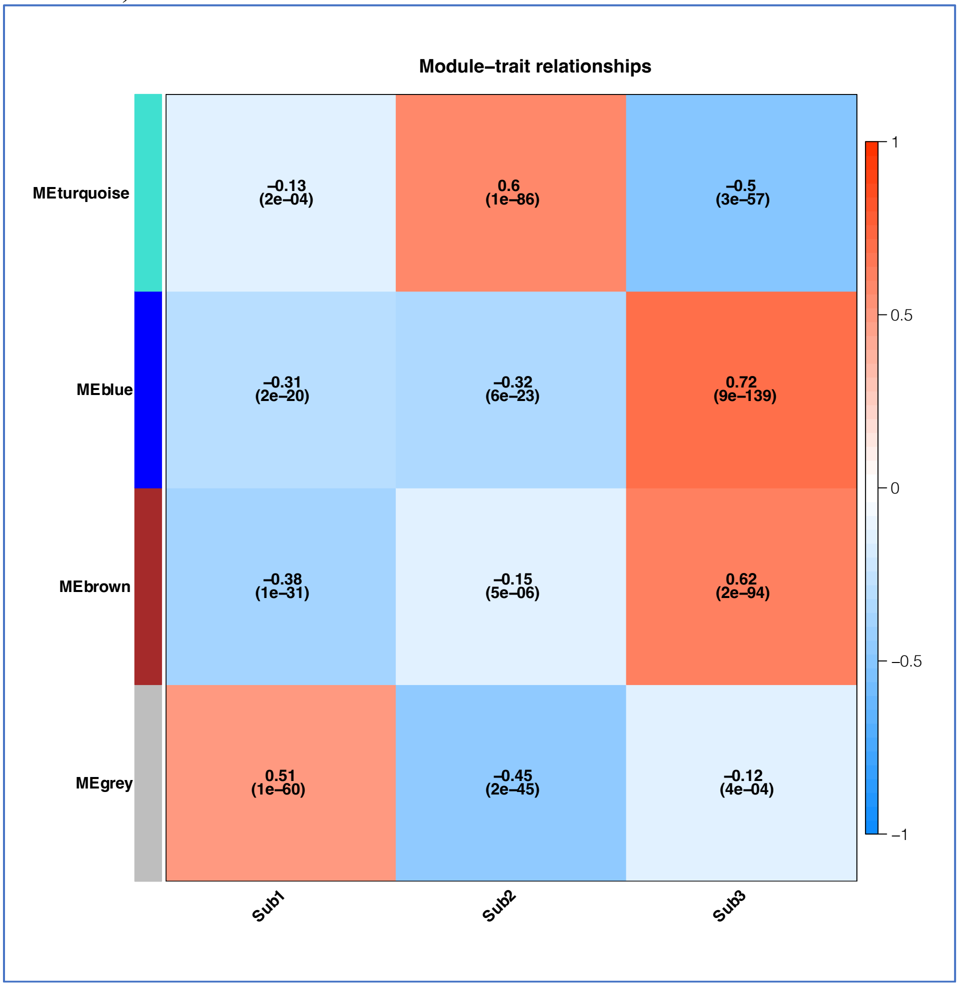

Supplementary Figure 6. Subgroup 2 shows a "*MYCN*" signature, potentially induced by Aurora Kinase A (AURKA) overexpression. Multivariate analysis of AURKA expression level and risk status in in *MYCN* non-amplified neuroblastomas. HR (hazard ratio), 95% CI (confidence interval), patient number (n), and *P* values are shown[35].

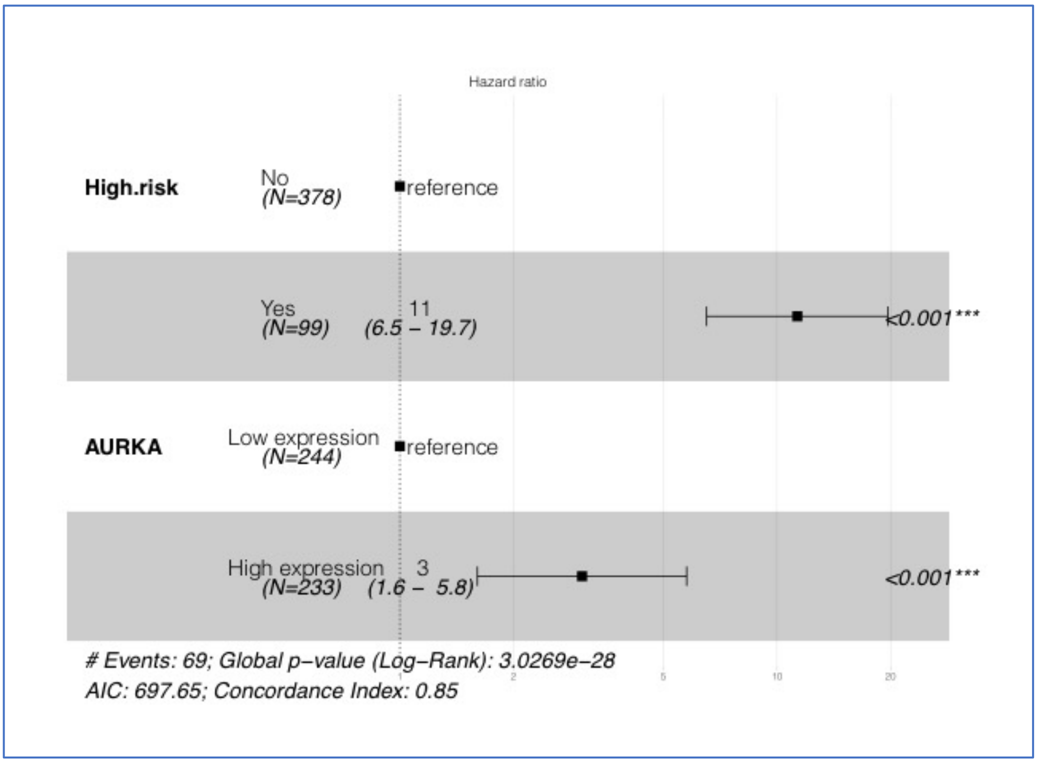

Supplementary Figure 7. Subgroup 3 is accompanied by an "inflamed" gene signature. (**a** and **b**) Violin plots showing stromal scores and tumour purity in different subgroups and *MYCN*-AMP in the train, test or train plus test cohort. (**c**) UMAP projection of *MYCN* non-amplified patients' cells. The colours demonstrated the distinct cell types according to the established marker genes. (**d**) SubMap analysis showing differential anti-PD1 immunotherapeutic response in 3 subgroups (GSE78220). Bonferroni adjusted *P* values indicated.

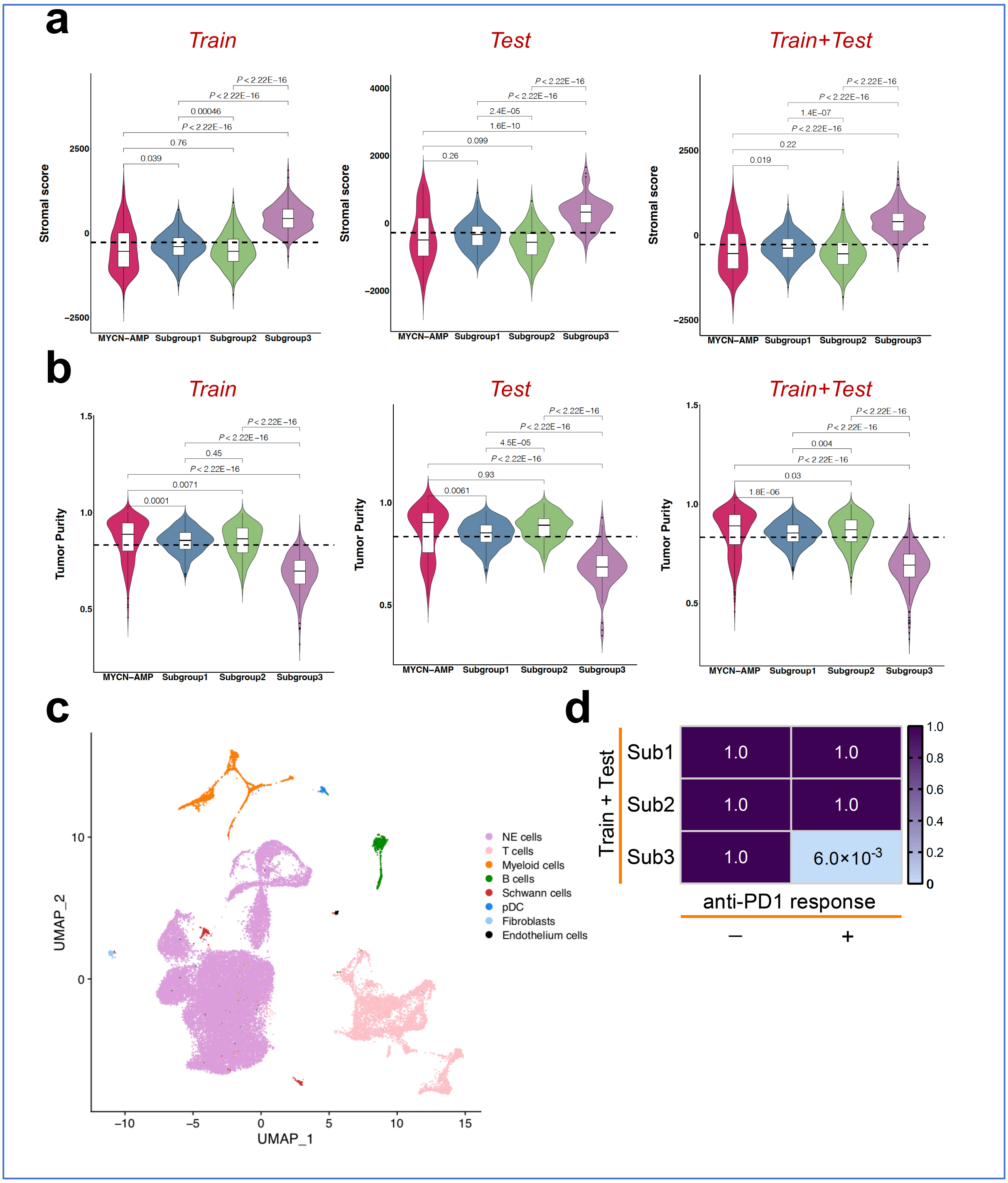

Supplementary Figure 8. Evaluation of different patient stratification strategies within *MYCN* non-amplified neuroblastomas. (**a** and **b**) Violin plots showing ADRN and MES scores in different subgroups and *MYCN*-AMP in train and test cohort. (**c**) Prediction differences in E-MTAB-1781 using subgrouping method from this report (named Hu) or Oberthuer and colleagues (Oberthuer's svm_th44). (**d**) Multivariate analysis of subgroup classification with Oberthuer's svm_th44 classification in *MYCN* non-amplified neuroblastomas. HR (hazard ratio), 95% CI (confidence interval), patient number (n), and *P* values are shown.

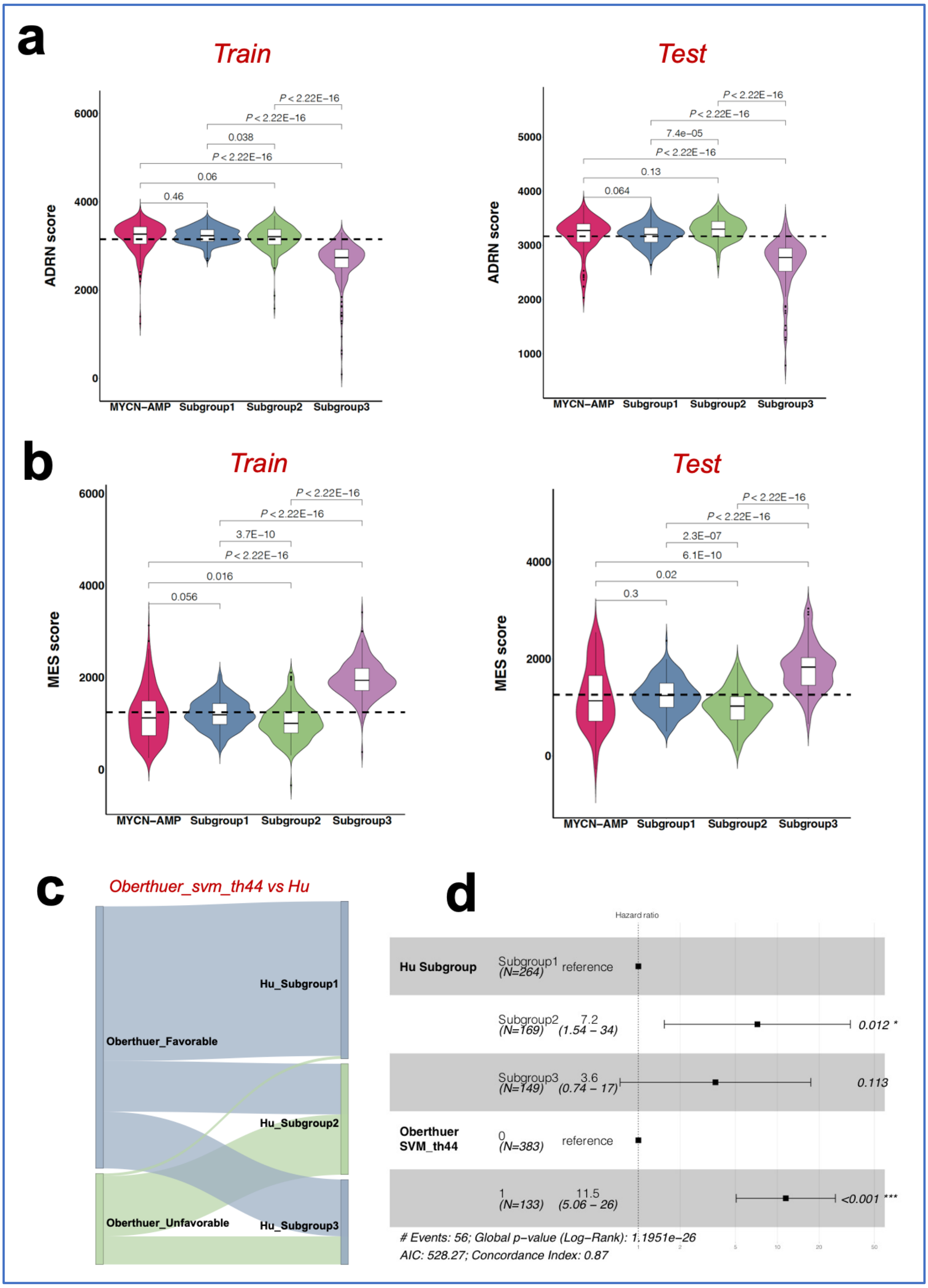

Supplementary Tables

Table S1. List of datasets and samples collected for meta-analysis.
Table S2. List of top 50% variable genes for consensus clustering.
Table S3. Univariate and multivariate regression analysis in *MYCN* non-amplified neuroblastomas.
Table S4. DEGs (differentially expressed genes) in subgroups.
Table S5. GSEA (gene set enrichment analysis) in subgroups.
Table S6. WGCNA (weighted gene co-expression network analysis) in subgroups.
Table S7. List of genes in PPI (protein–protein interaction) network analysis.
Table S8. IHC result from Xinhua hospital.
Table S9. List of 46 immune-related gene sets.
Table S10. List of predict genes and rules.
Table S11. Prediction results of multiple cohorts.
Table S12. Classification differences between different stratification strategies.
