# Supplementary material for: Identification of MYCN non-amplified neuroblastoma subgroups points towards molecular signatures for precision prognosis and therapy stratification
Source: Br J Cancer. Author manuscript; Available in PMC 2024 May 27. (PMC7616008; doi:10.1038/s41416-024-02666-y)
Supplement: Table S3 [file EMS194837-supplement-Table_S3.docx]

**Table S3**. Univariate and multivariate logistic regression analysis in *MYCN* non-amplified neuroblastomas.

|  | **OR (95% CI)** | **P-value** |
| --- | --- | --- |
| Univariate |  |  |
| ***Age*** |  |  |
| ≤ 18 months | **Reference** |  |
| > 18 months | 28 (15 ~ 54) | < 2.2E-16 |
| ***INSS Stage*** |  |  |
| Stage_1/2 | **Reference** |  |
| Stage_3 | 4.3 (1.84 ~ 10.00) | 7.72E-04 |
| Stage_4 | 19.3 (9.80 ~ 38.2) | < 2.2E-16 |
| Stage_4S | 1.3 (0.41 ~ 4.4) | 0.625 |
| ***Subgroup*** |  |  |
| Subgroup 1 | **Reference** |  |
| Subgroup 2 | 25.1 (12.2 ~ 52) | < 2.2E-16 |
| Subgroup 3 | 8.3 (3.8 ~ 18) | 1.03E-07 |

| Multivariate |  |  |
| --- | --- | --- |
| ***Age*** |  |  |
| ≤ 18 months | **Reference** |  |
| > 18 months | 14.8 (6.54 ~ 33.5) | 9.66E-11 |
| ***INSS Stage*** |  |  |
| Stage_1/2 | **Reference** |  |
| Stage_3 | 1.8 (0.77 ~ 4.4) | 0.17 |
| Stage_4 | 3.8 (1.82 ~ 7.8) | 3.42E-04 |
| Stage_4S | 4.1 (1.08 ~ 15.7) | 3.8E-02 |
| ***Subgroup*** |  |  |
| Subgroup 1 | **Reference** |  |
| Subgroup 2 | 6.6 (3.06 ~ 14.0) | 1.28E-06 |
| Subgroup 3 | 3..7 (1.69 ~ 8.3) | 1.14E-03 |

INSS: the International Neuroblastoma Staging System.
